# UV photocatalyzed polyurethane formulations for controlled foam generation

**DOI:** 10.1039/d6ra03376h

**Published:** 2026-08-11

**Authors:** Guillaume Cotte-Carluer, Leandro Jacomine, Hanieh Mansouri, Antoine Egelé, Damien Favier, Wiebke Drenckhan-Andreatta, Aurélie Hourlier-Fargette

**Affiliations:** a Université de Strasbourg, CNRS, Institut Charles Sadron UPR22 F-67000 Strasbourg France hourlierfargette@unistra.fr

## Abstract

Polymeric foams are used across a wide range of applications from insulation to tissue engineering. However, finely tuning their morphologies for property control remains challenging due to the difficulty of decoupling the foaming and the solidification. To achieve this decoupling, we use here an external stimulus to trigger the solidification of polyurethane foams. Our original strategy relies on the use of a photobase generator (OXTA-DBU), which, *via* the release of a cyclic amidine upon irradiation at the appropriate wavelengths, acts as a photolatent catalyst for the hydroxyl-isocyanate addition reaction. The response of the resulting bulk formulations to UV irradiation is characterized *via* the use of photorheology. Then, using a purpose-designed millifluidic circuit, these initially liquid formulations are continuously mixed and foamed. The obtained foam templates are solidified through UV irradiation. The structure of the resulting solid polyurethane foams is studied *via* X-ray microtomography to analyze the impact of irradiation conditions on the repartition of the polymeric matrix across the foam, providing a qualitative insight into the solidification kinetics of the foam. We thus demonstrate that the use of OXTA-DBU allows for the generation of well-controlled polyurethane foams whose final morphology can be adjusted through irradiation conditions. We also show that formulations mixing the photobase generator with a more classical catalyst provide access to an even wider range of solidification kinetics which, in turn, enables a finer tuning of the solid foam morphology.

## Abstract 2

By using a photobase generator as a latent catalyst, we achieve a UV-controlled solidification of polyurethane foams generated *via* millifluidics. The study combines photorheology on bulk polyol-isocyanates systems and foam morphology analysis.

## Graphical abstract

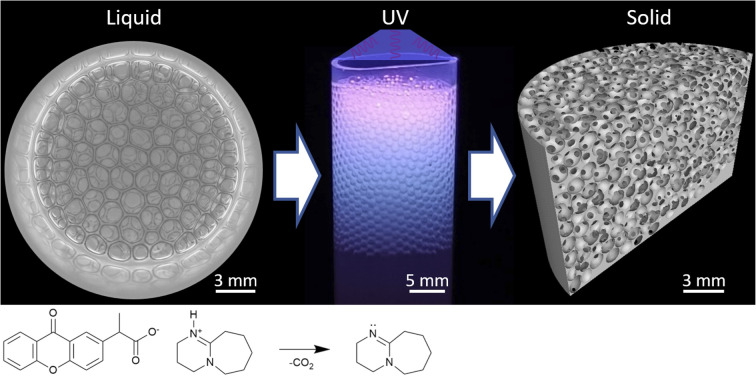

## Introduction

Liquid foam templating is an efficient strategy to generate solid foams with well-defined morphologies *via* a two-step process: (i) the discrete generation of liquid foams acting as templates and (ii) their subsequent solidification^1–3^, for example *via* controlled polymerization, gelation or crosslinking. However, only few examples of this approach in the context of polyurethane (PU) foams are described in the literature, despite PU foams being the most used polymeric foams in industry. This is most likely due to the fact that liquid foam templating requires the decoupling of the foaming and solidification processes which can prove difficult to achieve using polyol-isocyanate formulations.

Although previous studies demonstrated that this challenge can be overcome through the careful adjustment of the formulation reactivity^4^ or *via* the use of a secondary catalyst-rich foam draining into a less reactive liquid template,^5^ these methods lack flexibility or prove quite tedious. A more straightforward and versatile approach would be to rely on systems whose solidification can be controlled using an external stimulus. In particular, the use of UV light as a solidification stimulus has recently been demonstrated to allow for a fine control of the final foam structure with different polymeric materials. Visser and co-workers generated monodisperse foams by using acrylate^6,7^ as well as thiol–ene^8^ chemistry combined with radical photoinitiation to control the solidification of precisely deposited single bubbles. Similarly, foams with well-defined shapes were obtained by relying on the solidification of successive bubble layers^9,10^*via* the photoinitiated polymerization of acrylate-based systems. However, these processes work through partial solidification and as such do not allow for the full foam to be in a liquid state initially and to reach an “equilibrium structure” corresponding to the minimisation of interfacial energies in the system. On the other hand, Schüler *et al.*^11^ demonstrated that a monolithic foam prepared from a styrene emulsion containing a radical photoinitiator could be solidified using a UV curing step and a sintering step, therefore taking advantage of the controlled structure of the initial liquid template.

However, the solidification of the continuous matrix in these previous works relies on photoinitiated radical polymerization, and we wish to focus here on the UV-controlled polymerization of polyol-isocyanates PU. This choice of focusing on the use of polyol-isocyanate-based systems stems from the wide range of formulations allowing for the formation of stable liquid foams^4^ and yielding solid matrixes with a wide range of properties, building on almost 90 years of PU history.^12^ In terms of catalysis of such systems, different metallic complexes^13^ and organometallics containing tin^14,15^ or other metals^15^ have been shown to act as photolatent catalysts for the hydroxyl-isocyanate addition reaction in bulk polyurethane formation.

More recently, Zivic *et al.*^16^ demonstrated that photobase generators (PBG) releasing cyclic amidines can be used for the photopolymerization of polyol-isocyanate systems, thanks to the high catalytic efficiency of cyclic guanidines and amidines in the isocyanate–hydroxyl reaction.^17,18^ The use of organic PBG is particularly interesting when compared to organometallic alternatives due to the high toxicity of the latter, especially regarding the tin-based variants.

**Fig. 1:**
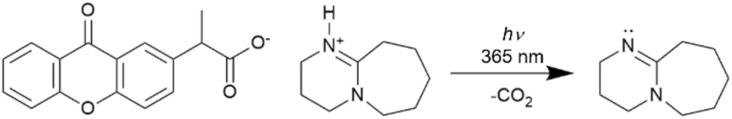
Activation of OXTA-DBU and release of DBU upon UV irradiation.

Here we design a polyol-isocyanate formulation for the generation of PU foams whose solidification can be controlled with UV light as a stimulus *via* the use of 2-(9-oxoxanthen-2-yl)propionic acid 1,8-diazabicyclo[5.4.0]-undec-7-ene salt (OXTA-DBU) (Fig. 1), a PBG that releases the cyclic amidine 1,8-diazabicyclo[5.4.0]-undec-7-ene (DBU) upon irradiation (Fig. 1). The choice of the OXTA-DBU/DBU couple for this study was motivated by the commercial availability of OXTA-DBU and by the fact that, among cyclic guanidines and amidines which are known to be very efficient catalysts for the addition reaction between hydroxyl and isocyanate groups,^18^ DBU was found by Alsarraf and co-workers^17^ to be especially efficient. We therefore conduct here a series of photorheological measurements to characterize the impact of UV irradiation on the solidification kinetics of formulations containing OXTA-DBU and/or the associated organic base DBU. We then take advantage of a home-designed millifluidic circuit combining static mixing and foaming of the selected formulations^19^ to generate monodisperse liquid foam templates. The impact of UV irradiation conditions on the solidification of these templates is characterized using X-ray microtomography to analyse the morphology of the obtained solid PU foams. By investigating the correlation between the solidification kinetics of bulk UV-sensitive formulations and the morphology of the foams generated from them, we optimise formulations containing OXTA-DBU alone and hybrid formulations containing both OXTA-DBU and DBU. We show that hybrid formulations are particularly promising candidates for externally triggered foam control since their solidification times are sufficiently short to supress foam ageing effects.

## Materials and methods

### Materials

The polyether-based triol *Lupranol 2095* (#OH = 35 mg KOH g^−1^) as well as the aliphatic polyisocyanate Basonat *HI 100 NG* (%NCO = 22%) based on hexamethylene diisocyanate isocyanurate were supplied by *BASF*. The silicone based surfactant *Tegostab B 8002* (#OH = 65 mg KOH g^−1^) was supplied by *Evonik*. OXTA-DBU (>98%) was purchased from TCI while DBU (98%) was purchased from Sigma-Aldrich. Lupranol was dried overnight *via* nitrogen bubbling (50 °C, 2 L h^−1^) before use, while the other species were used as received.

When preparing formulations containing OXTA-DBU, the poorly soluble photobase generator was dissolved in dichloromethane (99.8%, Carlo Erba) before being added to Lupranol. The dichloromethane was removed from the resulting solution before use *via* nitrogen bubbling (details are provided in the SI). Formulations containing OXTA-DBU were prepared in a dark room and protected from ambient light during mixing.

### Photorheology

The PU formulations used for photorheological measurements were prepared to obtain an isocyanate index of NCO/OH = 110% by weighing the A-component containing the polyol, the catalyst(s) and the surfactant (*ω*_surf_ = 2 wt%) (Tables S1 and S2), and the B-component containing the polyisocyanate alone, in two separate 10 mL syringes (*BBraun Injekt*). The two syringes were then connected with a section of *Tygon* PVC tubing (E-3603, 6.35 mm internal diameter) containing 5 elements of a helical static mixer (3M Scotch-Weld D48.5S Mixer Nozzle, 6.4 mm diameter, Fig. S1) and their content was blended by pushing the two pistons in an alternate manner for 5 min. An appropriate amount (≈0.16 mL) of the resulting PU formulation was then deposited in between the two rheometer geometries.

Photorheological measurements were performed using a Discovery HR-3 rheometer (*TA Instruments*) equipped with a UV light guide accessory (*TA instruments*). This accessory was connected *via* a 1 m light guide to an *Omnicure S2000 Elite* lamp equipped with a 365 nm filter (Fig. S2) which was used to irradiate the sample during its solicitation. A 20 mm quartz plate fixed to the light guide accessory served as the lower plate and a 40 mm stainless steel plate whose temperature was kept at 25 °C using the Upper Heating Plate system from *TA Instruments* served as the oscillating upper plate. The solidification kinetics of PU formulations were characterized by performing measurements as a function of time at 1 Hz using a geometry gap of *h* = 500 µm. The initial time *t* = 0 was set as the end of the formulation mixing. The time at which a sample was determined to have reached its gel point was the time *t*_c_ at which the measured storage modulus *G′* and loss modulus *G″* crossed each other.

For measurements performed in irradiated conditions, the irradiation was started when a first point was registered by the rheometer. Samples were irradiated with an irradiance of *E*_I_ = 300 mW cm^−2^ for a time Δ*t*_uv_ = 15 min.

### PU foam generation

**Fig. 2:**
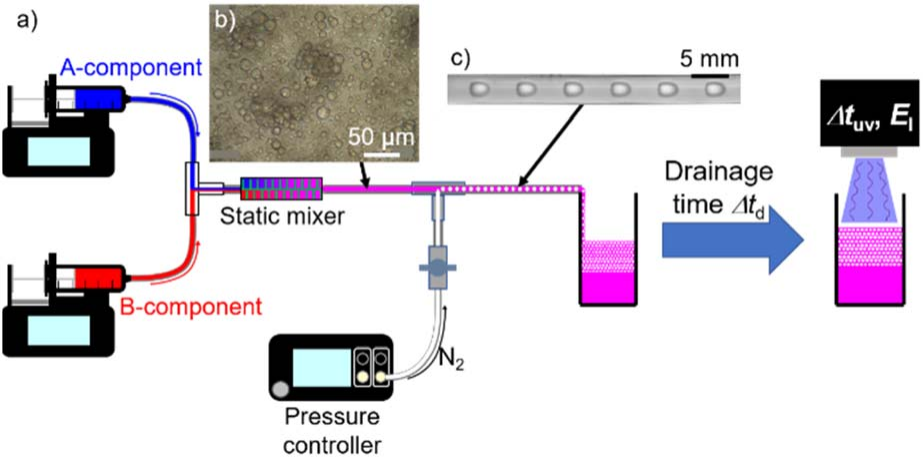
(a) Schematic drawing of the continuous mixing and foaming of PU formulations using a millifluidic circuit; (b) optical microscopy image (zoom x1000) of the emulsion of B-component in A-component generated by the static mixing module of the circuit; (c) photograph of the bubbles generated by the bubbling module of the circuit. After generation, the foam is left to settle for a time Δ*t*_d_ before being exposed to UV-light from the top for Δ*t*_uv_.

Liquid foam templates were generated by feeding the A-component and the B-component into a millifluidic circuit (Fig. 2a) at flow rates of respectively *Q*_A_ = 1 mL min^−1^ and *Q*_B_ = 0.12 mL min^−1^ to reach an Isocyanate Index of NCO/OH = 110% (Tables S1 and S2). Two *Standard Infusion Only Pump 11 Elite* Syringe Pumps (*Harvard Apparatus*) were used to do so.

Upon entering the circuit, the two components were blended using 50 mixing elements extracted from static mixers supplied by MedMix (T-mixer, 2.5 mm diameter, Fig. S3) which, as studied quantitatively in previous work^19^, enabled the continuous generation of a fine emulsion of B-component in A-component (Fig. 2b and S3) despite the high viscosity of the B-component (Table S3). Gas bubbles of controlled size (Bubble radius *R*_B_ ≈ 0.5 mm) were generated in this resulting PU formulation (Fig. 2c and S4) by using a T-junction with an internal diameter of 1.1 mm. This bubbling geometry was supplied with nitrogen at a constant pressure *P*_G_ = 230 mbar *via* an *Elveflow* pressure controller (*OB1 Mk3+*). The resulting bubbly formulation was collected at the outlet of the circuit in the lower half of a 10 mL polypropylene syringe (*BBraun Injekt*, 15.9 mm inner diameter) for a duration of Δ*t*_b_ = 5 min to build-up the liquid foams serving as templates.

The resulting liquid foams were then left to settle during a time Δ*t*_d_, before being irradiated at an irradiance of *E*_I_ = 300 mW cm^−2^ for an irradiation time Δ*t*_uv_.

### Microtomography characterization

Solid PU foams were scanned using an *EasyTom 150/160* X-ray microtomograph (*RX Solutions*) with a resolution of 14 µm per voxel. A 3D reconstruction of each scanned foam was built from the corresponding scan with the *X-Act* software (*RX Solutions*). Using the analysis software *VGStudio,* an image stack constituted of horizontal slices taken at regular vertical positions inside of the foam (every 14 µm) was extracted from the 3D reconstruction. By measuring the area fraction occupied by the solid PU matrix in each slice *via* an *ImageJ* macro (Macro S1), we obtained the evolution of the solid fraction *Φ*_S_ along the vertical axis *z* in the final solid foam.

This study of the foam morphology was conducted solely on the upper portion of the samples where *Φ*_S_ was measured to stay below the critical volume fraction *Φ*_C_ = 36% which corresponds to the jamming transition for monodisperse disordered foams, as this portion corresponds to a true foam and not to a mix of foam and bubbly bulk.

In addition, the strut thicknesses in the same region of interest were calculated using the “Foam and powder” tool from *VGStudio*.

## Results and discussion

### OXTA-DBU formulations

#### Bulk solidification kinetics

**Fig. 3:**
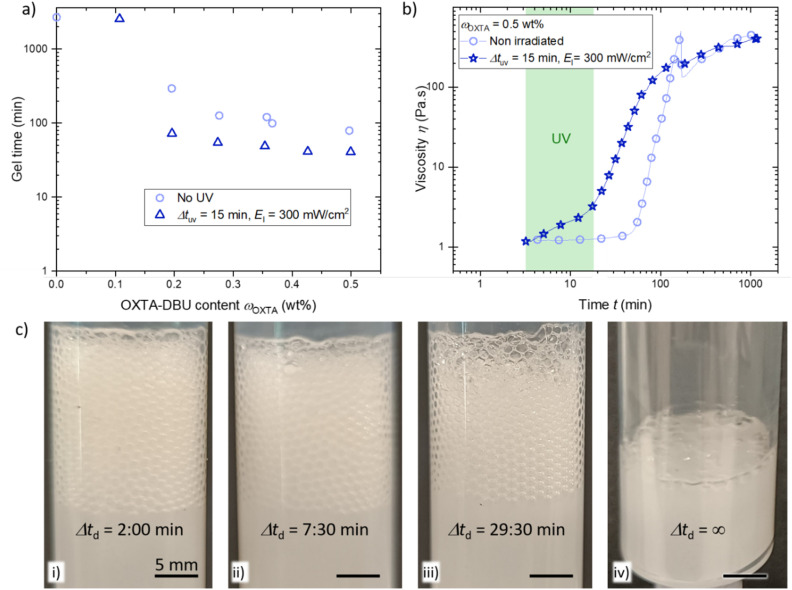
(a) Gel times *t*_c_ measured by photorheology at 25 °C, as a function of the OXTA-DBU content *ω*_OXTA_ for irradiated (Δ*t*_uv_ = 15 min, *E*_I_ = 300 mW cm^−2^) and non-irradiated bulk PU samples; (b) evolution of the viscosity *η*(*t*) as a function of the time *t* elapsed since the end of mixing, measured at *T* = 25 °C for irradiated and non-irradiated formulations with an OXTA-DBU content of *ω*_OXTA_ = 0.5 wt%; (c) photographs of solid PU foams obtained *via* irradiation of liquid templates after drainage times of (i) Δ*t*_d_ = 2:00 min, (ii) Δ*t*_d_ = 7:30 min, (iii) Δ*t*_d_ = 29:30 min and (iv) Δ*t*_d_ = ∞, 24 hours after their generation, with an OXTA-DBU content of *ω*_OXTA_ = 0.5 wt%.

At first, we characterized the solidification kinetics of formulations catalysed by OXTA-DBU alone and the impact of UV irradiation on said kinetics. To do so, the evolution of the visco-elastic shear moduli of formulations with increasing OXTA-DBU contents *ω*_OXTA_ were measured in irradiated (Δ*t*_uv_ = 15 min, *E*_I_ = 300 mW cm^−2^) and in non-irradiated conditions. The evolution of the elastic shear modulus G′ and the viscous loss modulus *G*″ are plotted in the SI (Fig. S5). The time *t*_c_ at the cross-over of the moduli (*G*′ = *G*″) is taken as the “gel point”. The obtained values are presented in Fig. 3a and examples of gelled samples observed after the separation of the rheometer plates are presented in Fig. S6 and reveal the apparition of bubbles within the process. Further work is needed to understand what drives this gas release and how to prevent it, however, based on the data shown in Fig. S6, we argue that those bubbles only have a limited effect on the measurement of *G′* and *G″* before *t*_c_ is reached. Formulations without catalyst have gel times of the order of 3000 minutes. When adding enough catalyst to the system (*ω*_OXTA_ ≥ 0.2 wt%), the solidification of the formulations accelerates in a significant manner, reducing their gel times in non-irradiated conditions and reducing it even further in irradiated conditions, causing them to reach 80 and 40 minutes, respectively, in the case of the formulation containing the highest OXTA-DBU concentration (*ω*_OXTA_ = 0.5 wt%). In addition, when looking at the evolution of the viscosity during sample solidification for a formulation containing *ω*_OXTA_ = 0.5 wt% (Fig. 3b), we are again able to discern a strong impact of UV irradiation on the solidification kinetics: while the viscosity stays almost constant for more than 30 min after the end of mixing in the non-irradiated sample, the viscosity of the irradiated sample shows a significant increase as soon as the irradiation is started. This difference in gel times and viscosity profiles between irradiated and non-irradiated formulations demonstrates that the OXTA-DBU contained in the PU formulation undergoes its activation reaction (Fig. 1) when exposed to UV light, therefore proving its relevance as a photolatent catalyst for PU formulations.

On the other hand, we observe that the addition of OXTA-DBU to the formulation decreases its gel time, even in non-irradiated conditions, proving that the inactivated photobase generator presents a non-negligible catalytic activity toward the polyol-isocyanate reaction. This undesired catalytic activity may stem from the presence of a secondary nitrogen atom in the DBU-H^+^ cation, that can act as a secondary catalytic site. However, it is also interesting to highlight the similarities between OXTA-DBU and other carboxylate-ammonium salts that are known to promote the formation of isocyanurate links *via* the cyclotrimerization of isocyanates.^20,21^

These measurements also show that, beyond a threshold concentration of approximately *ω*_OXTA_ = 0.28 wt%, the further addition of OXTA-DBU to the system only has a small impact on the solidification kinetics of the formulation, especially in irradiated conditions. This is most likely due either to the fact that the solubility limit of OXTA-DBU in the A-component may be reached around *ω*_OXTA_ = 0.28 wt% or to the limited penetration of UV in the emulsions constituting the PU formulations (Fig. S7 and S8).

#### Impact of UV irradiation on PU foam generation

Following the demonstration that UV irradiation can be used to adjust the solidification kinetics of PU formulations containing OXTA-DBU, we investigate its impact on the solidification of PU foam templates. Four liquid foam templates with bubble radii of *R*_B_ = 0.56 mm were generated from a formulation with an OXTA-DBU content of *ω*_OXTA_ = 0.5 wt% using the developed millifluidic circuit. The liquid templates were allowed to settle for increasing durations of Δ*t*_d_ = 2:00, 7:30, 29:30 min and Δ*t*_d_ = ∞ (non-irradiated sample) before being exposed to UV light (*E*_I_ = 300 mW cm^−2^) for Δ*t*_uv_ = 15 min. We refer to the time Δ*t*_d_ as “drainage time”, since during this time the foam essentially evolves *via* gravity-driven drainage of liquid towards the bottom of the foam. Other typical ageing mechanisms of foams, such as coalescence *via* film rupture or coarsening *via* gas exchange between bubbles, are initially supressed by the use of an efficient surfactant mixture and sufficiently large bubbles, respectively. Only if the foam is left to drain until loosing most of its liquid, it becomes sensitive to coalescence.

The resulting solid foams were photographed 24 hours later and are shown in Fig. 3c. The morphology, and more precisely the structural integrity of the obtained foam depends strongly on the irradiation conditions. Indeed, we observe that, for each sample, a fraction of the foam collapsed before its solidification, and that this fraction increases with increasing drainage times Δ*t*_d_. More specifically, no foam remains after solidification for Δ*t*_d_ = ∞ (Fig. 3c(iv)), and half of the foam collapsed before solidifying for Δ*t*_d_ = 29:30 min (Fig. 3c(iii)). On the other hand, only the top 4–5 layers and 2–3 layers of bubbles were destroyed for the foams obtained after Δ*t*_d_ = 7:30 min (Fig. 3c(ii)) and Δ*t*_d_ = 2:00 min (Fig. 3c(i)), respectively. This observation can be explained by the fact that with increasing drainage time Δ*t*_d_, the liquid content in the foam decreases with the lowest liquid content being at the top of the foam. For a very low liquid content, the surfactants are much less efficient in protecting the bubbles from coalescence, creating a progressive rupture of the foam from the top.

The characteristic drainage time of a liquid foam^22^ scales as
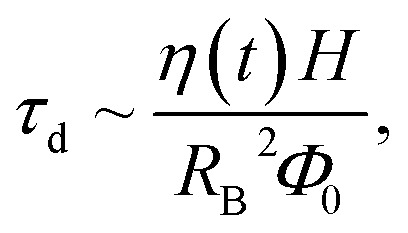
where *η*(*t*) is the viscosity of the liquid, which evolves with time, *H* is the height of the foam, *R*_B_ the bubble size and *Φ*_0_ the initial liquid content of the foam. Since *H*, *R*_B_ and *Φ*_0_ are identical for all samples, the drainage time is essentially influenced by the change in viscosity. In non-irradiated foams without catalyst, drainage significantly impacts the foam only during the first 15 minutes following the end of the template generation (inset in Fig. 6a). Hence, a measurable increase of the viscosity before 15 minutes, which is the case in irradiated samples containing catalyst with Δ*t*_d_ = 2:00 min and 7:30 min, will have a significant impact on the foam stability.

Hence, we can conclude here on a strong impact of the irradiation conditions on the structural integrity of solid PU foams obtained *via* the solidification of otherwise identical liquid templates. As in the case of bulk samples, we see that the solidification kinetics of PU foams containing OXTA-DBU can be controlled using UV light as a stimulus.

However, we also see that despite the high catalyst concentrations, the evolution of the viscosity is slow (Fig. 3b) and the gel times remain measurably longer than the characteristic drainage times. Since increasing the catalyst concentration is not an option due to the plateau in the gel times (Fig. 3a) we therefore propose in the following to work with hybrid formulations mixing OXTA-DBU and DBU in an effort to reduce further the gel times.

### Hybrid formulations

#### Bulk solidification kinetics

Following the conclusion that OXTA-DBU alone cannot be used to achieve gel times below 40 minutes, even in irradiated conditions, we decided to introduce in the formulation the amidine released by OXTA-DBU, *i.e.* DBU, to act as a more traditional catalyst, in order to gain access to a wider range of solidification kinetics.

**Fig. 4:**
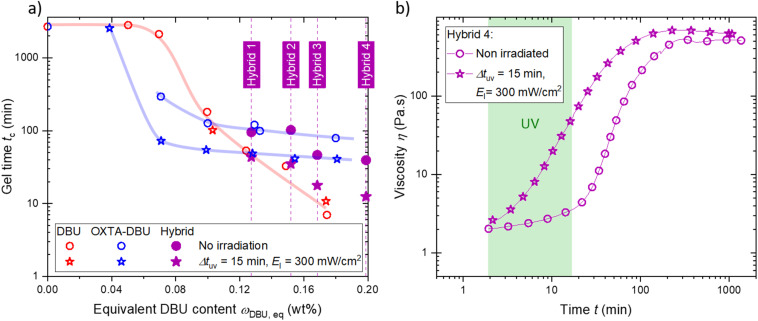
(a) Gel times *t*_c_ measured by photorheology at 25 °C, as a function of the equivalent DBU content *ω*_DBU,eq_ for irradiated (Δ*t*_uv_ = 15 min, *E*_I_ = 300 mW cm^−2^) and non-irradiated PU bulk samples catalysed by: DBU alone (red), OXTA-DBU alone (blue), and hybrid systems (purple) (Table S2, Hybrid 1: *ω*_OXTA_ = 0.28 wt% and *ω*_DBU_ = 0.025 wt%, Hybrid 2: *ω*_OXTA_ = 0.28 wt% and *ω*_DBU_ = 0.05 wt%, Hybrid 3: *ω*_OXTA_ = 0.24 wt% and *ω*_DBU_ = 0.083 wt%, Hybrid 4: *ω*_OXTA_ = 0.28 wt% and *ω*_DBU_ = 0.1 wt%); (b) evolution of the viscosity *η* as a function of the time *t* elapsed since the end of mixing, measured at *T* = 25 °C for irradiated and non-irradiated hybrid formulations with an equivalent DBU content *ω*_DBU,eq_ = 0.2 wt% (Hybrid 4: *ω*_OXTA_ = 0.28 wt% and *ω*_DBU_ = 0.1 wt%).

As such, we started by quantifying the impact of DBU on the solidification kinetics of the PU formulation. The gel times of formulations containing increasing amounts of DBU were measured in non-irradiated conditions and are presented by red circles in Fig. 4a alongside the gel times measured previously for OXTA-DBU alone (blue circles and stars). The full evolutions of the visco-elastic moduli are given in Fig. S9 in the SI. It must be noted that, in order to simplify the comparison between the different catalytic systems, all the gel times presented in Fig. 4a are plotted as a function of the equivalent DBU content *ω*_DBU,eq_. The equivalent DBU content *ω*_DBU,eq_ corresponds to the total DBU content which is expected to be found in a formulation for which the entirety of the contained OXTA-DBU underwent its activation reaction. The equivalent DBU content of a formulation is calculated from the initial DBU and OXTA-DBU contents, *ω*_DBU_ and *ω*_OXTA_ respectively, using the following formula:
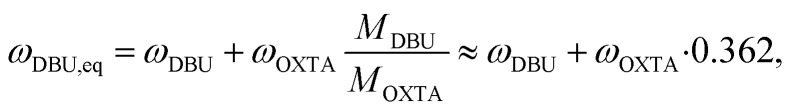
with *M*_DBU_ (=152.24 g mol^−1^) and *M*_OXTA_ (=420.5 g mol^−1^) being the molar mass of the two catalysts.

These measurements, presented in Fig. 4a, show that, for low DBU concentrations (*ω*_DBU,eq_ ≤ 0.07 wt%), the addition of DBU to the formulation does not measurably impact the gel time. This is most likely due to the fact that, as a strong organic base, DBU can easily react with residual water, leading to the formation of a far less reactive conjugated acid. Even though the formulations where dried beforehand (see Materials and Methods), environmental humidity enters rapidly into the thin samples on the rheometer and can therefore not be avoided. However, once enough DBU has been added to the system to neutralize the water in the sample (*ω*_DBU,eq_ ≥ 0.1 wt%), the formulation gel times decrease roughly exponentially with increasing DBU content, reaching values as low as *t*_c_ = 7 min for *ω*_DBU,eq_ = 0.175 wt%.

Two additional gel time measurements performed on pure DBU formulations in irradiated conditions (red stars in Fig. 4a, and S10) show that the solidification kinetics of formulations containing DBU alone are not significantly impacted by UV irradiation. This observation supports the conclusion drawn in the previous section, according to which the difference between the gel times of irradiated and non-irradiated samples catalysed by OXTA-DBU are caused by its activation under UV light.

In the following, we investigate the solidification kinetics of hybrid formulations containing both DBU and OXTA-DBU. In order to profit from maximum photosensitivity, the hybrid formulations were prepared with OXTA-DBU contents of approximately *ω*_OXTA_ = 0.28 wt% (Table S2), as it corresponds to the beginning of the plateau in the gel time reported in the previous section for formulations catalysed by OXTA-DBU only. Different DBU contents *ω*_DBU_ are then added to this base formulation in order to vary to equivalent DBU content *ω*_DBU,eq_ (Table S2). The resulting measurements (purple circles and stars in Fig. 4a and S11) show that for DBU contents below *ω*_DBU_ = 0.05 wt% (*ω*_DBU,eq_ ≤ 0.15 wt%) the measured gel times, in irradiated and non-irradiated conditions, are very close to the ones obtained for formulations catalysed by OXTA-DBU only. This can be explained by the low quantities of DBU added to the system which might be entirely neutralized by residual water. On the other hand, if enough DBU is added to the system to overcome the presence of residual water (*ω*_DBU,eq_ ≥ 0.17 wt%), the gel times in both irradiated and non-irradiated conditions shorten significantly.

Despite an acceleration of the solidification kinetics in both conditions, a significant gap between irradiated and non-irradiated samples can be observed, thus proving that the tested formulations keep their photosensitive properties.

This difference between the irradiated and non-irradiated hybrid formulations can also be observed when following the evolution of the viscosity *η* during the solidification of formulations containing *ω*_OXTA_ = 0.28 wt% and *ω*_DBU_ = 0.1 wt% (*ω*_DBU,eq_ = 0.2 wt%, “Hybrid 4”) in irradiated and non-irradiated conditions (Fig. 4b). Indeed, the viscosity *η* of the irradiated sample reaches 10 Pa s in less than 10 min while it takes more than 30 min for the one of the non-irradiated sample to reach a similar value. This demonstrates the relevance of such hybrid systems for applications where short but adjustable solidification kinetics are needed.

On the other hand, contrary to what was observed previously for OXTA-DBU catalysed formulations (Fig. 3b), *η* increases significantly from the start of the measurements, even in non-irradiated conditions. This difference in behaviours between OXTA-DBU catalysed and hybrid formulations must be highlighted as it will most likely impact the foam generation and the ageing of the generated foams.

#### Impact of UV irradiation on foam morphology

Liquid foam templates with *R*_B_ = 0.49 mm were generated from the formulation with the fastest solidification kinetics in irradiated conditions (*ω*_OXTA_ = 0.28 wt% and *ω*_DBU_ = 0.1 wt% → *ω*_DBU,eq_ = 0.2 wt%, “Hybrid 4”). Out of the four generated liquid foams, three were irradiated after Δ*t*_d_ = 2:15 ± 0:05 min at *E*_I_ = 300 mW cm^−2^ for Δ*t*_uv_ = 1, 5, and 10 min respectively and one was kept in the dark until solidification (Δ*t*_uv_ = 0).

The resulting foams show a key difference from the ones obtained with formulations catalysed by OXTA-DBU only: all of the hybrid foams retain their integrity during solidification, without evidence of bubble coalescence, even in the case of the non-irradiated foam.

**Fig. 5:**
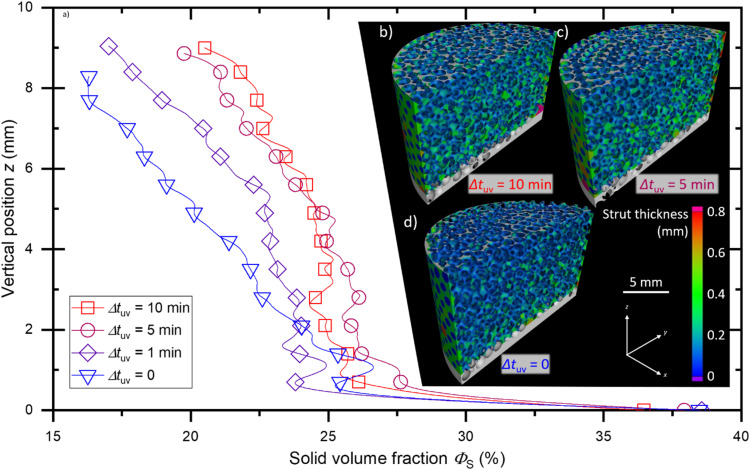
(a) Evolution of the solid fraction *Φ*_S_ as a function of the vertical position *z* in solid PU foams generated from the “Hybrid 4“ formulation (Table S2, *ω*_DBU,eq_ = 0.2 wt%: *ω*_OXTA_ = 0.28 wt% and *ω*_DBU_ = 0.1 wt%), and irradiated after Δ*t*_d_ = 2:15 ± 0:05 min for Δ*t*_uv_ = 0, 1, 5, and 10 min; (b–d) 3D reconstructions obtained by microtomography and colour graded according to the local strut thickness for the solid PU foams for (a) Δ*t*_uv_ = 10 min, (b) Δ*t*_uv_ = 5 min, and (c) Δ*t*_uv_ = 0.

This is due to the fact that the presence of DBU leads to a measurable increase in viscosity and a progressive solidification even in the absence of irradiation, reducing sufficiently the impact of drainage in order to prohibit coalescence. On the other hand, the profile of the solid fraction *Φ*_S_ differs significantly between the final foams (Fig. 5a). Globally, all profiles correspond to those of foams whose gravity-driven drainage has been arrested at some point:^22,23^ the initially liquid foam with a homogeneous liquid fraction loses progressively its liquid towards the bottom, with the bottom liquid fraction being fixed at 36% for randomly packed, equal-volume spherical bubbles. Stronger drainage leads to less steep liquid (and hence solid) fraction profiles, which is consistent with the observations done in previous work.^19,24^ We see that foams irradiated for Δ*t*_uv_ = 5 and 10 min show similar profiles with rather high solid content, while shorter irradiations times Δ*t*_uv_ result in lower solid fractions and less steep profiles. This observation demonstrates that templates subjected to a longer irradiation solidify quicker, stopping the drainage occurring in the liquid foam earlier, resulting in higher solid fraction *Φ*_S_ in the final solid PU foam.

We can also conclude from the similar morphologies of the Δ*t*_uv_ = 5 and 10 min foams that only the first 5 minutes of the irradiation matter, most likely because, beyond this point, the viscosity *η* of the liquid matrix becomes high enough to stop the foam drainage until its solidification. This difference between the *Φ*_S_ profiles of the different foams can also be observed when analysing their strut thicknesses (Fig. S12), plotted in Fig. 5b–d. The colour-graded 3D reconstructions show that the struts constituting the foam obtained for Δ*t*_uv_ = 0 are noticeably thinner than the ones obtained for Δ*t*_uv_ = 5 and 10 min which have more similar morphologies. This is also reflected by the calculated average strut thickness 〈*e*_S_〉 which is measurably lower in the foam obtained for Δ*t*_uv_ = 0 (〈*e*_S_〉 = 0.23 mm) than in the one obtained for Δ*t*_uv_ = 10 min (〈*e*_S_〉 = 0.28 mm).

In conclusion, the time over which the foam is exposed to the UV irradiation provides control over the final foam morphology, and, in the case of our foam samples and irradiation intensities, the irradiation time reaches a limit of influence for durations beyond 5 min. In the following we therefore use an irradiation time of Δ*t*_uv_ = 5 min to investigate the influence of the drainage time on the final foam morphology.

**Fig. 6:**
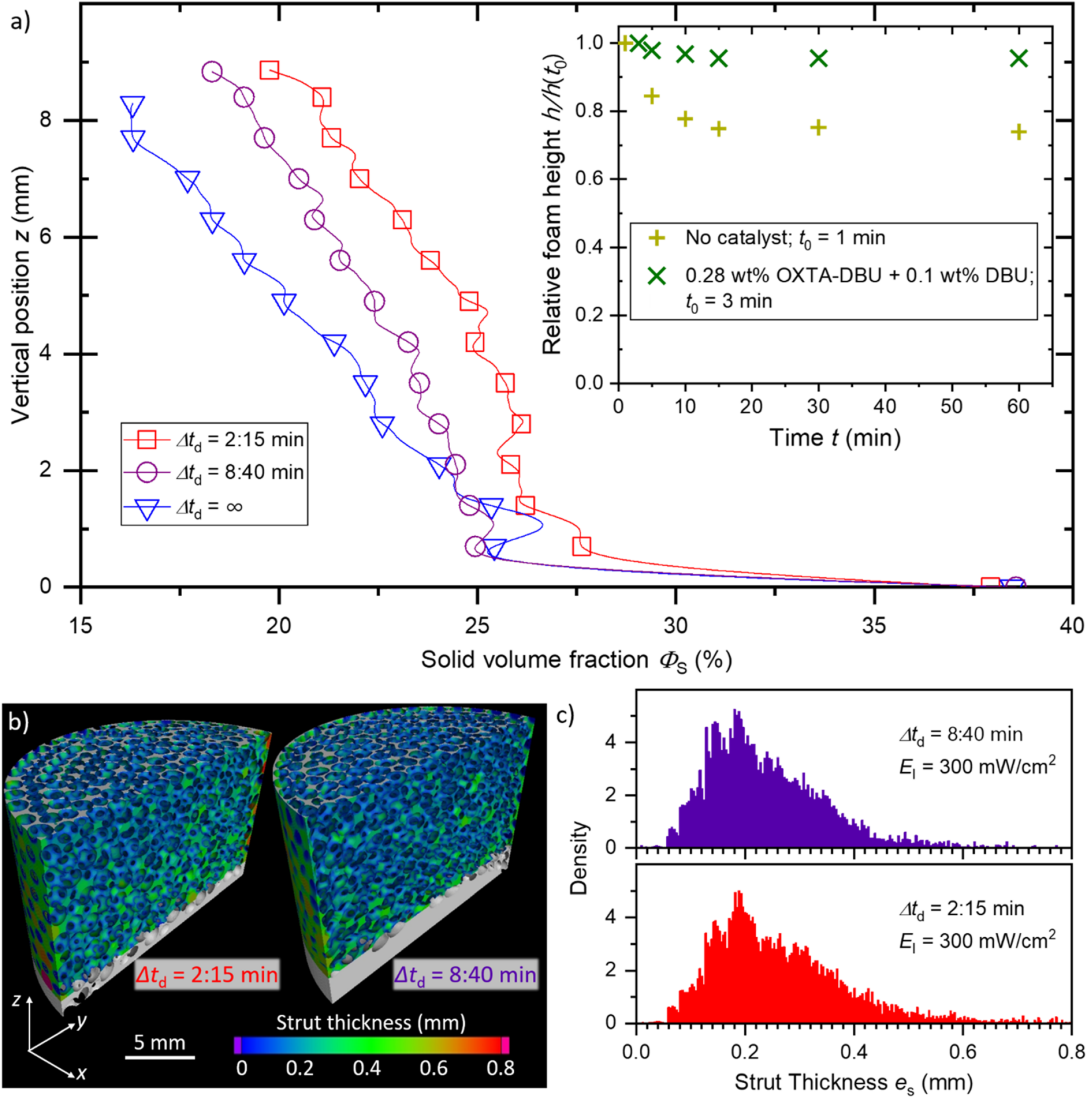
(a) Evolution of the solid fraction *Φ*_S_ as a function of the vertical position *z* in solid PU foams generated from the “Hybrid 4” formulation (Table S2, *ω*_DBU,eq_ = 0.2 wt%: *ω*_OXTA_ = 0.28 wt% and *ω*_DBU_ = 0.1 wt%), and irradiated for Δ*t*_uv_ = 5 min after drainage times of Δ*t*_d_ = 2:15 min, Δ*t*_d_ = 8:40 min, and Δ*t*_d_ = ∞; ((a) Inset) evolution, as a function of the time *t* elapsed since the end of foaming, of the foam height normalised by the initial foam height measured after *t* = *t*_0_ for a catalyst-free foam and for a foam prepared from the “Hybrid 4” formulation containing *ω*_OXTA_ = 0.28 wt% and *ω*_DBU_ = 0.1 wt%; (b) 3D reconstructions obtained by microtomography and colour graded according to the local strut thickness for the solid PU foams generated from the “Hybrid 4” formulation and irradiated after Δ*t*_d_ = 2:15 min and Δ*t*_d_ = 8:40 min, using the distribution of the strut thicknesses *e*_S_ calculated by *VGStudio* in the same PU foams, shown in (c).

In Fig. 6a we compare the *Φ*_S_ profiles of 2 hybrid foams irradiated for Δ*t*_uv_ = 5 min after Δ*t*_d_ = 2:15 min and Δ*t*_d_ = 8: 40 min with the *Φ*_S_ profile of a non-irradiated hybrid foam (Δ*t*_d_ = ∞) with the same formulation as used in the previous paragraph. We observe significant morphology differences between the three foams. For the foams irradiated after shorter Δ*t*_d_, the irradiation of the liquid template causes its solidification and therefore the cessation of drainage earlier, resulting in an overall higher solid fraction *Φ*_S_. This observation is supported by monitoring the evolution of the foam height in the liquid foam templates, which is plotted in the inset of Fig. 6a for formulations with and without catalyst in the absence of irradiation. The total foam height being composed of the total liquid and gas content, the change in foam height – in the absence of bubble coalescence – is entirely due to liquid draining out of the foam. We notice that both liquid templates cease to evolve only 15 min after the end of foam generation. Therefore, we conclude that the generated liquid templates are subjected to drainage for 15 min after the end of their generation, and that any change to the rheological properties of the formulation during those first 15 min will impact the morphology of the resulting solid foam.

The impact of an early irradiation on drainage results in solid foams with thicker struts as can be seen in the colour-coded 3D reconstructions (Fig. 6b and S13) and in the corresponding strut thickness distributions (Fig. 6c and S13). The foam obtained *via* the irradiation of the template after Δ*t*_d_ = 2:15 min presents overall thicker struts (〈*e*_S_〉 = 0.26 mm) than the ones constituting the foam obtained while using a drainage time of Δ*t*_d_ = 8:40 min (〈*e*_S_〉 = 0.24 mm).

In addition, we can observe that the template generated from a hybrid formulation containing *ω*_OXTA_ = 0.28 wt% and *ω*_DBU_ = 0.1 wt% (*ω*_DBU,eq_ = 0.2 wt%) loose less of its original height when compared with the one generated from a catalyst-free formulation. This is due to the viscosity increase that was observed to occur in hybrid formulations even in the absence of irradiation (Fig. 4b). Indeed, this early increase, which we attribute to the presence of a classical catalyst (DBU) in the formulation, slows down the drainage of the liquid template right after its generation, therefore limiting its overall impact on the final foam structure, which in turn limits the range of morphologies that can be accessed by adjusting the irradiation conditions.

In addition to the foams presented in this section, we also irradiated a liquid template after a drainage time of Δ*t*_d_ = 2:10 min for Δ*t*_uv_ = 5 min but using an irradiance of *E*_I_ = 600 mW cm^−2^ instead of *E*_I_ = 300 mW cm^−2^. The solid fraction profile of the resulting foam is shown in the SI in Fig. S14 and demonstrates that the intensity of the irradiation also impacts significantly the system solidification kinetics and that there is an interest, in the context of future studies, to quantify more systematically the impact of *E*_I_ on the final foam morphology.

In conclusion, hybrid formulations allow to push the gel times sufficiently down to (1) obtain solid PU foams with full structural integrity, and (2) tune measurably the final foam morphology *via* the choice of the irradiation conditions and the moment of their application.

## Conclusions

This work demonstrates that a photobase generator releasing cyclic amidines upon irradiation (OXTA-DBU) can be used as UV photolatent catalyst to control the solidification kinetics of bulk polyurethane formulations, as well as the solidification of the foams generated from them. Using gel times and viscosity measurements performed *via* photorheology, we showed that the curing speed of bulk PU formulations could be tuned through UV irradiation conditions. We also demonstrated that the use of hybrid formulations, containing both OXTA-DBU and the corresponding cyclic amidine DBU, widens the range of solidification kinetics that can be achieved. In addition, with the help of microtomography measurements, we were able to show that depending on the formulation and on the irradiation conditions, the structural integrity or the internal morphologies could be tuned, in the context of liquid templates generated using millifluidics.

Considering the limited penetration depth of UV light in foams due to absorption and scattering, the use of UV as external trigger will certainly concentrate on applications with one small sample dimensions (foam sheets or fibres for 3D-printing, for example).

Although we were able to access a wide range of solidification kinetics while retaining the photosensitive properties of formulations by using both DBU and OXTA-DBU, it would also be interesting to investigate in future work how the use of different monomers impacts the system behaviour. More specifically, relying on more reactive monomers or monomers with higher functionalities could enable the resulting formulation to reach shorter gel times in irradiated conditions while avoiding the early viscosity rises that were observed in non-irradiated hybrid formulations. This could in turn enable one to use the equations describing foam ageing kinetics to compute the irradiation parameters needed to achieve the desired solid foam morphology.

Additionally, future studies focusing on the UV-induced heating of the formulation and its impact on the solidification kinetics would be of a great interest to gain a more complete understanding and mastery of the developed formulations and of their curing kinetics. However, achieving the decoupling between thermal effects and the action of UV on the photobase generator – which is necessary to study this topic more quantitatively – might prove difficult in the case of foamed systems, in particular due to the insulating properties of polymer foams. Therefore, focusing on bulk systems would constitute a first step to consolidate the understanding of the systems at hand. It could, for example, be of great interest to study *via* photorheology the solidification kinetics of samples in adiabatic conditions, or to monitor the rheological properties of such samples subjected to temperatures profiles similar to the ones that can be measured at the core of the generated foams during their irradiation (Fig. S15).

## Author contributions

Guillaume Cotte-Carluer: investigation, writing – original draft, writing – review and editing, visualization, methodology, validation, data curation; Leandro Jacomine: methodology, resources, writing – review and editing; Hanieh Mansouri: investigation, visualization; Antoine Egelé: methodology, resources, visualization; Damien Favier: methodology, resources; Wiebke Drenckhan: conceptualization, writing – original draft, writing – review and editing, supervision, project administration, funding acquisition; Aurélie Hourlier-Fargette: conceptualization, writing – original draft, writing – review and editing, visualization, supervision, project administration, funding acquisition.

## Conflicts of interest

There are no conflicts to declare.

## Supplementary Material

RA-OLF-D6RA03376H-s001

RA-OLF-D6RA03376H-s002

## Data Availability

Additional data (rheological and tomographic characterization) can be found in the supplementary information (SI). Supplementary information is available. See DOI: https://doi.org/10.1039/d6ra03376h. A video illustrating the 3D reconstruction, *via* X-ray microtomography, of a solid PU foam (Fig. 5b, “Hybrid 4” formulation, Δ*t*_uv_ = 10 min, Δ*t*_d_ = 2:20 min) was also submitted alongside the present article. Additional data can be requested from the authors.

## References

[cit1] Andrieux S., Drenckhan W., Stubenrauch C. (2018). Langmuir.

[cit2] Andrieux S., Quell A., Stubenrauch C., Drenckhan W. (2018). Adv. Colloid Interface Sci..

[cit3] Stubenrauch C., Menner A., Bismarck A., Drenckhan W. (2018). Angew. Chem., Int. Ed..

[cit4] Testouri A., Ranft M., Honorez C., Kaabeche N., Ferbitz J., Freidank D., Drenckhan W. (2013). Adv. Eng. Mater..

[cit5] Jouanlanne M., Egelé A., Drenckhan W., Farago J., Hourlier-Fargette A. (2024). Macromol. Rapid Commun..

[cit6] Rastogi P., Venner C. H., Visser C. W. (2022). Polymers.

[cit7] Visser C. W., Amato D. N., Mueller J., Lewis J. A. (2019). Adv. Mater..

[cit8] Amato D. N., Amato D. V., Sandoz M., Weigand J., Patton D. L., Visser C. W. (2020). ACS Appl. Mater. Interfaces.

[cit9] Weber P., Cai L., Rojas F. J. A., Garciamendez-Mijares C. E., Tirelli M. C., Nalin F., Jaroszewicz J., Święszkowski W., Costantini M., Zhang Y. S. (2024). Aggregate.

[cit10] Cheng D. Y., Tai W. C., Liao Y. C. (2024). ACS Appl. Mater. Interfaces.

[cit11] Schüler F., Schamel D., Salonen A., Drenckhan W., Gilchrist M. D., Stubenrauch C. (2012). Angew. Chem., Int. Ed..

[cit12] Bayer O. (1947). Angew. Chem..

[cit13] DeVoe R. J. and Lynch D. C., US 4, 740, 577, 1988

[cit14] Van Den Berg K. J. , Van Oorschot J. C., Benningshof-Hulsbos E. and Van Beelen J. C., WO 2007/147851 A1, 2007

[cit15] Carroy A., Hintermann T., Baudin G., Bauer D., Contich P., Dietliker K., Faller M., Kohli Steck R., Lordelot C., Misteli K. (2010). Prog. Org. Coat..

[cit16] Zivic N., Sadaba N., Almandoz N., Ruipérez F., Mecerreyes D., Sardon H. (2020). Macromolecules.

[cit17] Alsarraf J., Ammar Y. A., Robert F., Cloutet E., Cramail H., Landais Y. (2012). Macromolecules.

[cit18] Sardon H., Pascual A., Mecerreyes D., Taton D., Cramail H., Hedrick J. L. (2015). Macromolecules.

[cit19] Cotte-Carluer G. , PhD Thesis, Université de Strasbourg, 2025

[cit20] Peyrton J., Avérous L. (2021). Mater. Sci. Eng., R.

[cit21] Salamone J. C. , Polymeric Materials Encyclopedia, CRC Press, Taylor & Francis Group, 2020, vol. 9

[cit22] Cantat I. , Cohen-Addad S., Elias F., Graner F., Höhler R., Pitois O., Rouyer F., Saint-Jalmes A., Flatman R. and Cox S., Foams : Structure and Dynamics, Oxford University Press, Incorporated, 2013

[cit23] Weaire D. L. and Hutzler S., The Physics of Foams, Clarendon Press, 2005

[cit24] Cotte-Carluer G., Egelé A., Jacomine L., Drenckhan-Andreatta W., Hourlier-Fargette A. (2026). Extreme Mech. Lett..

