# Supplementary material for: UV photocatalyzed polyurethane formulations for controlled foam generation
Source: RSC Adv. 2026 Aug 11;16(40):46798–807. doi: 10.1039/d6ra03376h (PMC13459182; doi:10.1039/d6ra03376h)

Supplementary Information (SI) for RSC Advances.
This journal is © The Royal Society of Chemistry 2026

UV photocatalyzed polyurethane formulations for controlled foam generation

Guillaume Cotte-Carluer,[a] Leandro Jacomine, [a] Hanieh Mansouri, [a] Antoine Egelé, [a] Damien Favier, [a] Wiebke Drenckhan-Andreatta [a] and Aurélie Hourlier-Fargette* [a]

Université de Strasbourg, CNRS, Institut Charles Sadron UPR22, F-67000, Strasbourg, France.
E-mail: hourlierfargette@unistra.fr

Supplementary Information

Table S1: Composition of the DBU and OXTA-DBU catalysed formulations whose gel times were measured via photorheology (percentages given in reference to the total mass of A-component). The A- and B-component constituting the OXTA-DBU catalysed formulation (ω_{OXTA}= 0.5 wt%) which was used for the generation of PU foams were also prepared using the concentrations detailed in this table.

A-Component m_A = 10 g		
	DBU catalysed	OXTA-DBU catalysed
Lupranol 2095	97.825-98 wt%	97.5-98 wt%
DBU	0-0.175 wt%	
OXTA-DBU		0-0.5 wt%
Tegostab B8002	2 wt%	
B-component (*NCO/OH* = 110%) m_B = 1.33 g		
Basonat HI 100 NG	13.3 wt%	

Table S2: Composition of the hybrid formulations whose gel times were measured via photorheology (percentages given in reference to the total mass of A-component). The A- and B-component constituting the "Hybrid 4" formulation ($\omega_{DBU,eq}$ = 0.2 wt%) which was used for the generation of PU foams were also prepared using the concentrations detailed in this table.

A-Component ($\omega_{DBU,eq}$ = 0-0.2 wt%) m_A = 10 g				
	Hybrid 1	Hybrid 2	Hybrid 3	Hybrid 4
Lupranol 2095	97.695 wt%	97.67 wt%	97.677 wt%	97.62 wt%
OXTA-DBU	0.28 wt%	0.28 wt%	0.24 wt%	0.28 wt%
DBU	0.025 wt%	0.05 wt%	0.083 wt%	0.1 wt%
Tegostab B8002	2 wt%			
B-component (*NCO/OH* = 110%) m_B = 1.33 g				
Basonat HI 100 NG	13.3 wt%			

Preparation of formulations for photorheology measurements

Firstly, 10 g of the A-component containing the polyol and the different additives are prepared by weighting appropriate amounts (Table S1, Table S2) of: Lupranol, Tegostab, 0.5 wt% DBU in Lupranol solution, and OXTA-DBU in Lupranol solution, in a 10 mL syringe (BBraun, Injekt). In order to prepare the OXTA-DBU in Lupranol solution, a 0.05 g/mL solution of OXTA-DBU in dichloromethane is prepared by weighting the two species in a 4 mL vial. The solution is then homogenized for at least 10 min using a magnetic stirrer before being drawn using a 1 mL glass syringe (*S.G.E. Gas Tight Luer Lock Syringe, SUPELCO*). The appropriate volume of the OXTA-DBU formulation is subsequently added to a weighted amount of Lupranol. The resulting polyol-dichloromethane-photocatalyst solution is homogenized for 10 min using a magnetic stirrer to guarantee an even dispersion of the OXTA-DBU in the medium. The solution is then subjected to a two-step evaporation process to eliminate the dichloromethane from the system. Firstly, the solution is kept under vacuum at room temperature for roughly 15 min, before being bubbled through with nitrogen using two needles, at a flow rate of Q_{N2} = 0.5 L/min for approximately 24 h, while also being kept in a bain-marie at 40 °C and being stirred using a magnetic stirrer. Once the evaporation step completed, the resulting OXTA-DBU in Lupranol solution is degassed under vacuum for roughly 15 min in order to remove the remaining nitrogen bubbles, before finally being weighted in the 10 mL syringe alongside the other species.
The B-component which consists of Basonat alone is prepared by weighting the appropriate amount (Table S1, Table S2) of the polyisocyanate in a second 10 mL syringe.
The two syringes containing the A- and B-components are linked together using 5 helicoidal mixing elements extracted from a *3M Scotch-Weld D48.5S Mixer Nozzle* (Figure S1**.**a). Once the two syringes are linked, their pistons are pushed by hand in an alternate fashion **(**Figure

S1.b) for 5 minutes. Once the mixing is done, the formulation is pushed into one of the syringes which is then disconnected from the mixing elements and the second syringe. The formulation contained in this syringe is then ready for use.

Figure S1: a) Photograph of 5 helicoidal static mixer elements extracted from a 3M Scotch-Weld D48.5S Mixer Nozzle and used in the double-syringe set-up to improve the mixing quality; b) Illustration of the double-syringe process coupled with the use of static mixers, used to mix A and B-components.

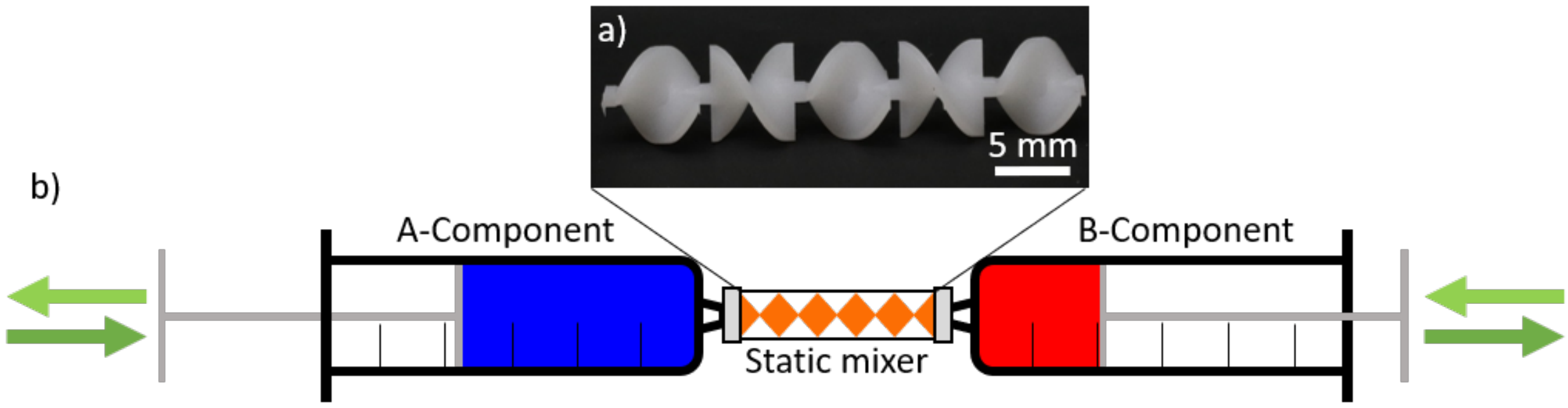

Preparation of formulations for liquid foam templating

Firstly, an OXTA-DBU in Lupranol solution containing twice the desired final OXTA-DBU content (Table S1, Table S2) is prepared in a 100 mL vial. To do so, OXTA-DBU is dissolved in a minimum amount of dichloromethane in said vial. Upon complete dissolution of OXTA-DBU in the solvent, Lupranol is added to the solution and, after 15 min of vigorous homogenization via the use of a magnetic stirring bar, the dichloromethane is removed from the system via a two-step drying process. Firstly, the solution is placed under vacuum at room temperature for at least 15 min. Secondly, nitrogen is bubbled at a flow rate of Q_{N2} = 2 L/min through the solution using fritted glass. This bubbling is performed for at least 24 hours while keeping the vial in a bain-marie at 40 °C and using a magnetic bar to stir the solution. The resulting OXTA-DBU in Lupranol solution is then degassed for 15 min under vacuum.

Subsequently, a Lupranol based formulation containing appropriate amounts of DBU (Table S1, Table S2) and Tegostab (4 wt%) is added to the OXTA-DBU in Lupranol solution. When mixing these two solutions equal masses of each are used. The resulting A-component is then homogenized for 3 hours using a magnetic stirrer before being transferred in a 100 mL glass syringe (*IPAS Interchangeable*).

It must be noted that argon gas is introduced inside the vial during the different preparation steps (excepted during the drying and degassing steps), as well as inside of the syringe, in order to limit the A-component contamination.

Similarly, the B-component is prepared by drawing Basonat inside of a 30 mL glass syringe (*Micro-Mate Interchangeable, Popper*) and argon gas is again used to limit contamination.

The syringes containing both components are put onto *Only Pump 11 Elite* Syringe Pumps (*Harvard Apparatus*) which are used to feed the millifluidic circuit.

Figure S2: Emission spectrum acquired using a Flame-S spectrometer (Ocean Optics) of the mercury lamp + filter system (Omnicure Elite S2000 + 365 nm filter) used for the irradiations performed during this study. The measured spectrum presents a main emission peak at 365 nm as well as a secondary peak at 335 nm.

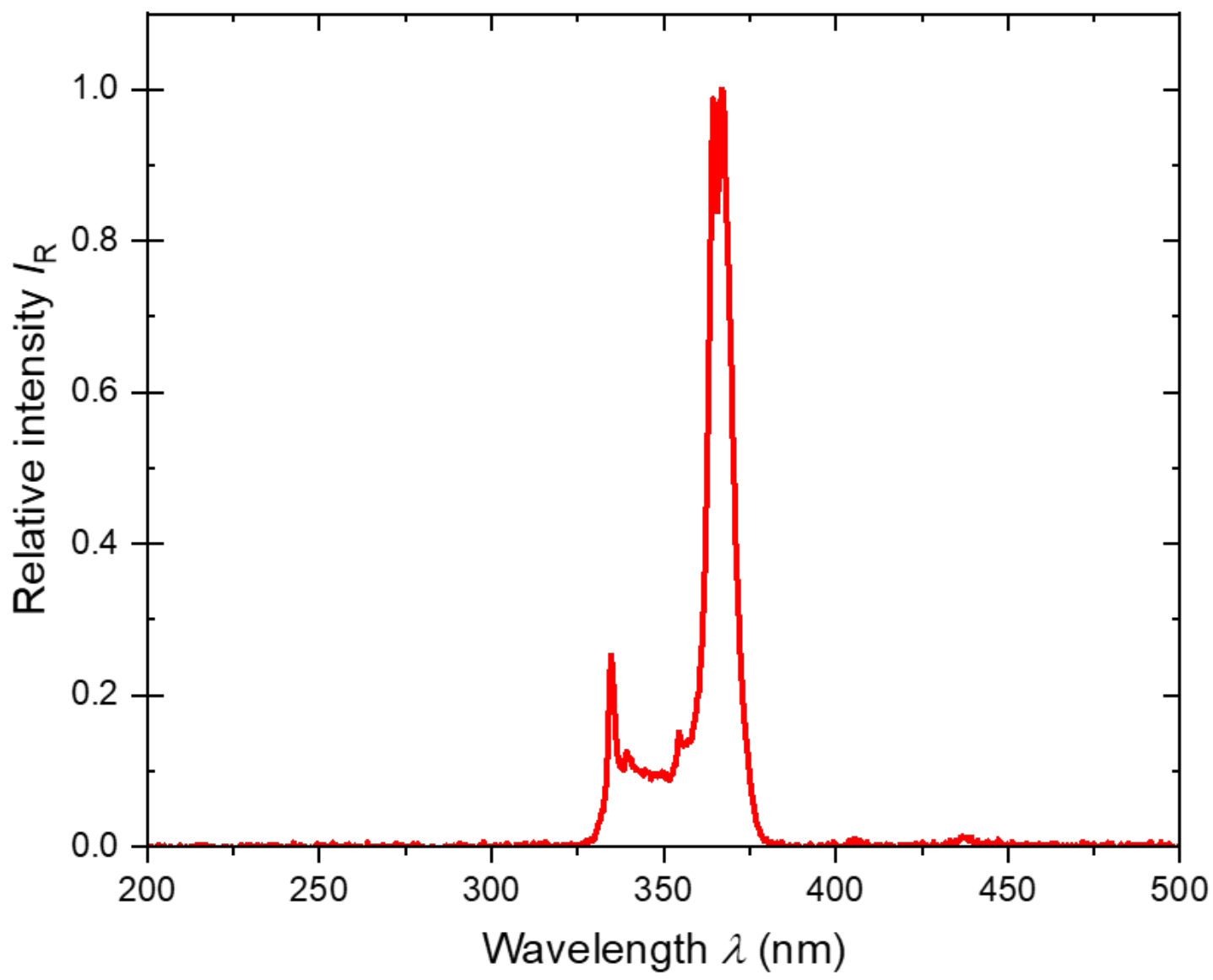

Figure S3: a) Photographs of one of the 2.5 mm T-mixer assemblies (10 mixing elements) provided by MedMix which are used in the millifluidic mixing module; b, c) Photographs illustrating the preparation of the millifluidic module: b) Firstly, 5 mixing assemblies are slid into PVC tubing (*Tygon E-3603*) with an internal and external diameters of 2.38 mm and 3.97 mm respectively, c) the tubing containing the mixers is then slid in a 4 mm cylindric hole drilled through a polycarbonate block in order to force the liquid flow through the mixing elements by preventing the tube from expand under pressure; d) Schematic drawing of the mixing module integration in the millifluidic set-up; e) Optical microscopy image acquired using a *VHX-X1* microscope (*Keyence*) of a catalyst-free emulsion (Table S1) generated by the mixing module (Q_A = 1 mL/min, Q_B = 0.12 mL/min). This image shows that the designed mixing module enables the generation of fine B- in A-component emulsions constituted of droplets with radii inferior to 15 µm despite the fact that the B-component has a higher viscosity than the A-component (Table S3).

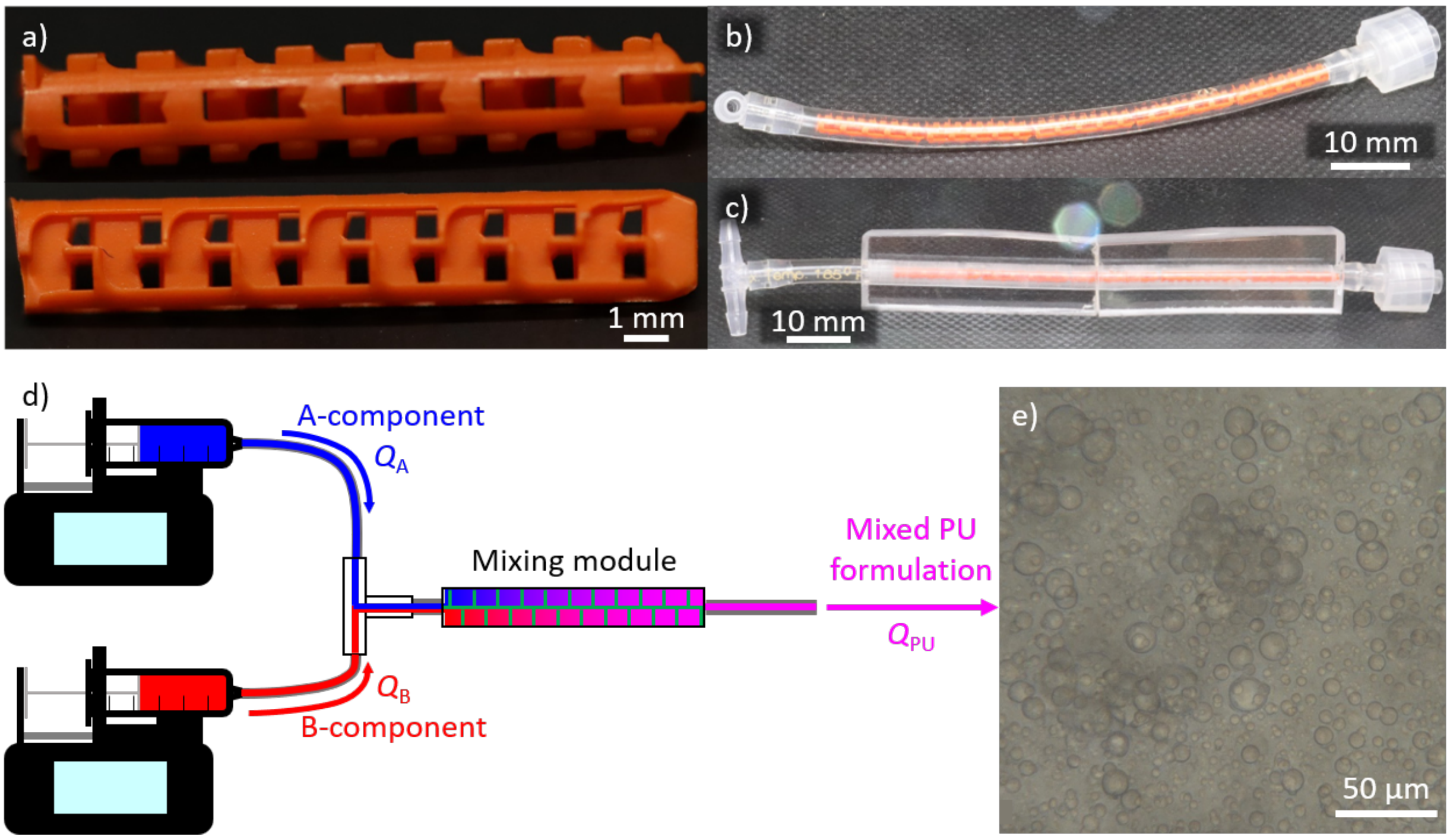

Table S3: Viscosities at 20 °C of the different individual species needed to generate a polyurethane formulation and of the corresponding A-component. Those viscosities were measured by performing a frequency sweep from f = 1 Hz to f = 800 Hz using a 40 mm cone geometry and a Pelletier plate to control the temperature.

Compound	Viscosity η (Pa.s)
Lupranol 2095	1.14
Tegostab B8002	0.8
A-component	1.14
Basonat HI 100 NG	3.840

Figure S4: a) Schematic drawing of the bubbling module integration in the millifluidic set-up; b) Photographs taken with a *uEye* camera (*IDS*, *UI-3580LE-C-HQ*) of the bubbles traveling in the PVC tubing (*Tygon E-3603*, 1.59 mm internal diameter) linking the T-junction to the foam collection set-up; c) Evolution over time of the average area of the bubbles being generated in hybrid formulations with equivalent DBU content of $\omega_{DBU,\,eq}$ = 0.175 wt% ("Hybrid 3") and $\omega_{DBU,\,eq}$ = 0.2 wt% ("Hybrid 4") (Table S2). These measurements demonstrate that the developed millifluidic device enables stable bubbling in reactive formulations which in turn allow for the generation of monodisperse liquid templates.

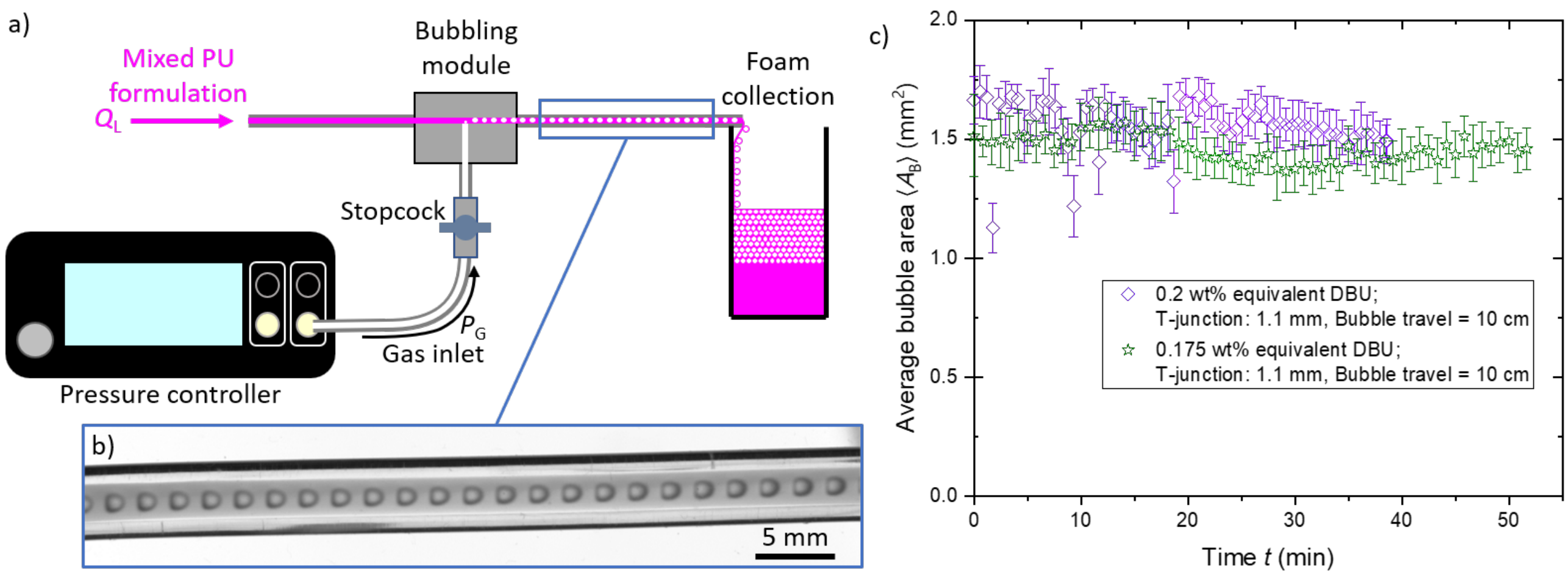

Macro S1: ImageJ macro used to calculate the vertical solid fraction Φ_S profile in a PU foam from an image stack of horizontal sections of said foam:

```
dir1 = getDirectory("C:/Image Stack path");          #
list = getFileList(dir1);                            #
setBatchMode(true);                                  # Selection of the image folder and successive
for (i=0; i<list.length; i++) {                      # opening of the different images in the stack
path = dir1+list[i];                                 #
open(path);                                          #

run("8-bit");                                        #
run("Threshold...");                         # Binarization of the opened
setOption("BlackBackground", true);                  # image to separate voids
setThreshold(50, 255);                               # from the polymer matrix
run("Convert to Mask");                              #

makeOval(540, 190, 900, 900);                        # Define the region of interest (here the)

run("Measure");                                      #

selectWindow("Results");                             #
saveAs("Results", dir1 + "Results file path");       # Saving of the result file
close("Results");                                    # and closing the windows
close("Threshold");                          # related to the macro
close("log");                                        #
```

For this macro to function as intended, the “Area fraction” box must be check in the “Set Measurements” menu of *ImageJ*.

Figure S5: Evolution of the storage modulus *G′* and loss modulus *G′′* measured via photorheology for formulations with increasing OXTA-DBU contents (Table S1) in non-irradiated and irradiated conditions. The results show that, for $\omega_{OXTA} \geq 0.2$ wt%, *G′* and *G′′* rise and cross each other sooner than in catalyst-free systems, even in non-irradiated systems. We observe that the irradiation of the sample further accelerates its solidification. These measurements also demonstrate that, for $\omega_{OXTA} \geq 0.28$ wt%, the further addition of OXTA-DBU to the system does not impact *G′* and *G′′* profiles in non-irradiated conditions nor in irradiated conditions.

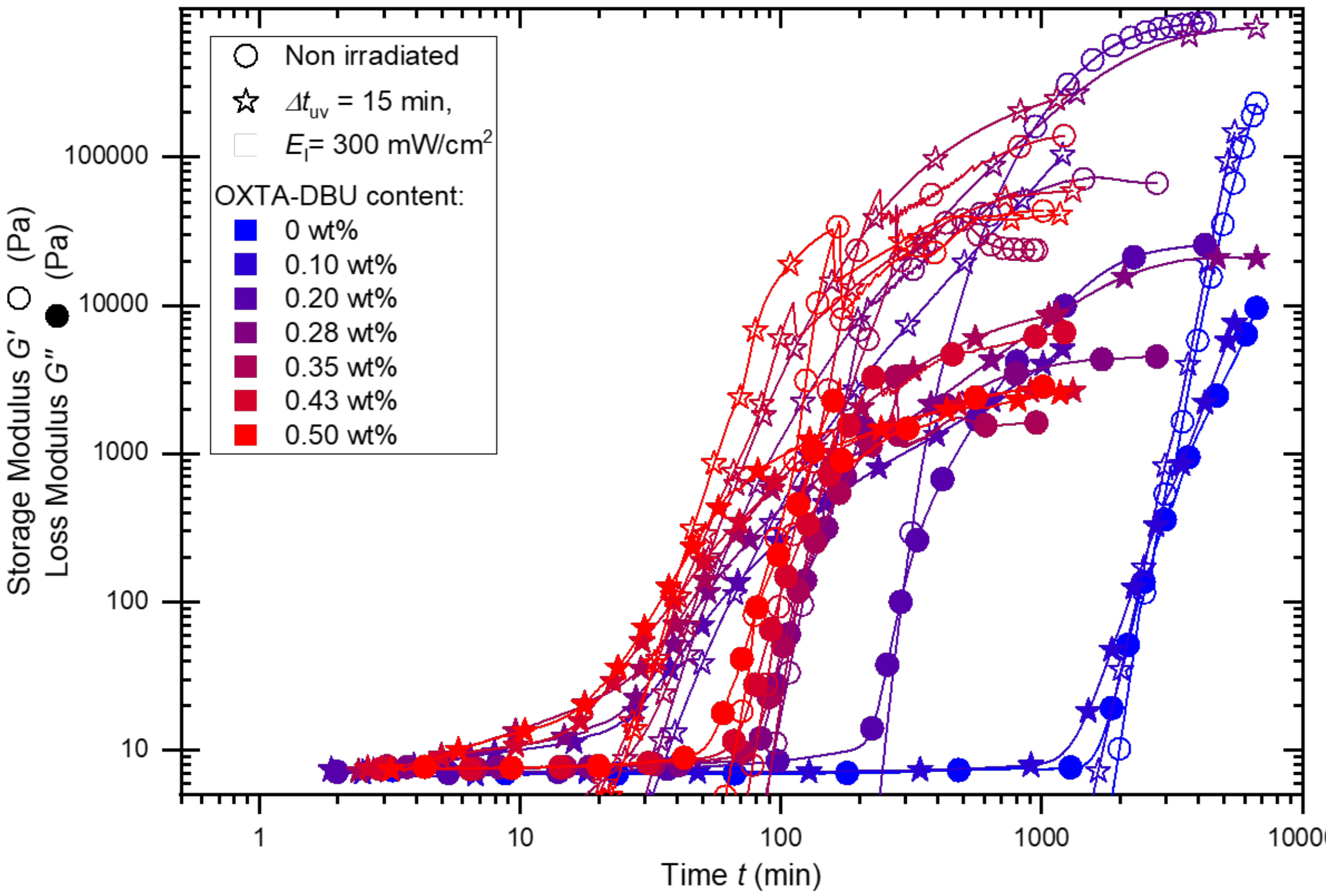

Figure S6: a) Photograph of the solid PU sample observed by separating the rheometer's plates after the measurement performed for the formulation containing ω_{OXTA} = 0.5 wt%; b-d) Evolution of the geometry gap h separating the rheometer's plates during the gel time measurements, plotted as a function of the time elapsed since the end of sample mixing t normalized by the sample gel time t_c, for irradiated (stars) and non-irradiated (circles) samples prepared using b) DBU catalysed (Table S1), c) OXTA-DBU catalysed (Table S1) , and d) hybrid (Table S2) formulations. The results show that, during some of the gel time measurements, bubbles are generated inside of the sample and apply pressure onto the rheometer's plates, causing the geometry gap h to increase as the gel time measurement procedure was designed to keep the axial force exerted on the sample constant. Although the mechanisms driving the generation of those bubbles is still not well understood and further work is needed to prevent their apparition, two trends emerge from these measurements: formulations containing OXTA-DBU and formulations with shorter gel times are prone to greater gap increases.

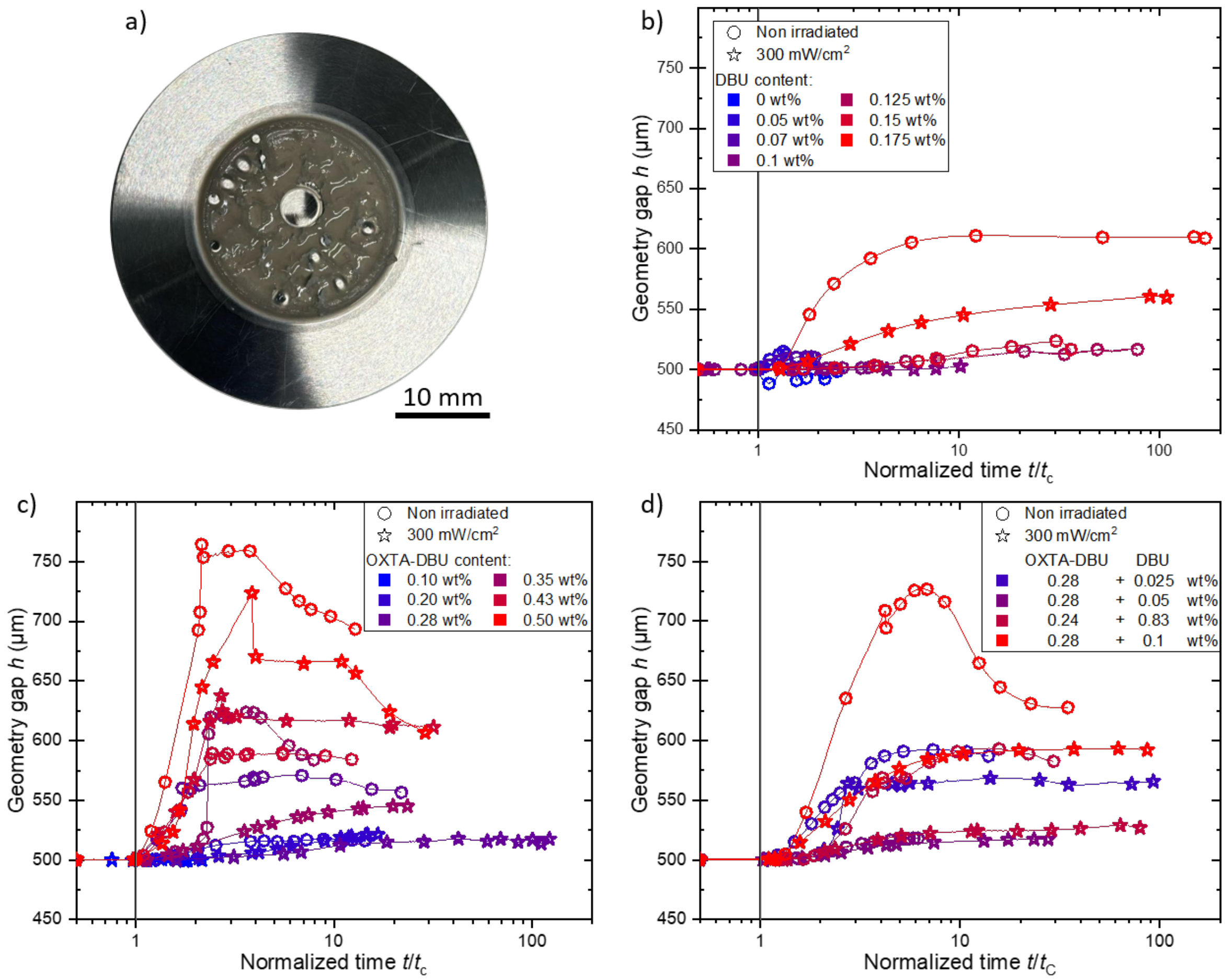

Figure S7: a) Transmission spectra of A-components containing no catalyst (blue line) and ω_{OXTA} = 0.5 wt% of OXTA-DBU (Table S1) (red line), measured on ℓ = 500 μm samples using a *Cary 5000 UV-Vis-NIR* Spectrophotometer (*Agilent*) equipped with a *Cary Universal Measurement Accessory* (*Agilent*); b) Absorption spectra for OXTA-DBU in dichloromethane formulations of decreasing concentrations measured on ℓ = 10 mm samples using a *Lambda 25* spectrophotometer (*Perkin Elmer*). The measurements show that, while UV light penetrates easily in A-components containing no OXTA-DBU, the penetration of UV light in A-components containing OXTA-DBU is greatly reduced for the wavelengths corresponding to the emissions peaks shown in Figure S2 due to the strong absorption coefficient of OXTA-DBU around λ = 346 nm which disturbs the penetration of UV light in the sample for wavelengths below 380 nm. The penetration is especially weak at λ = 335 nm, where the measured transmission I/I_0 is close to 0 for the formulation containing ω_{OXTA} = 0.5 wt%. On the other hand, at λ = 335 nm where OXTA-DBU has a lesser absorption coefficient, I/I_0 = 50.8 wt% meaning that half of the initial intensity is able to penetrates through the ℓ = 500 μm thick sample.

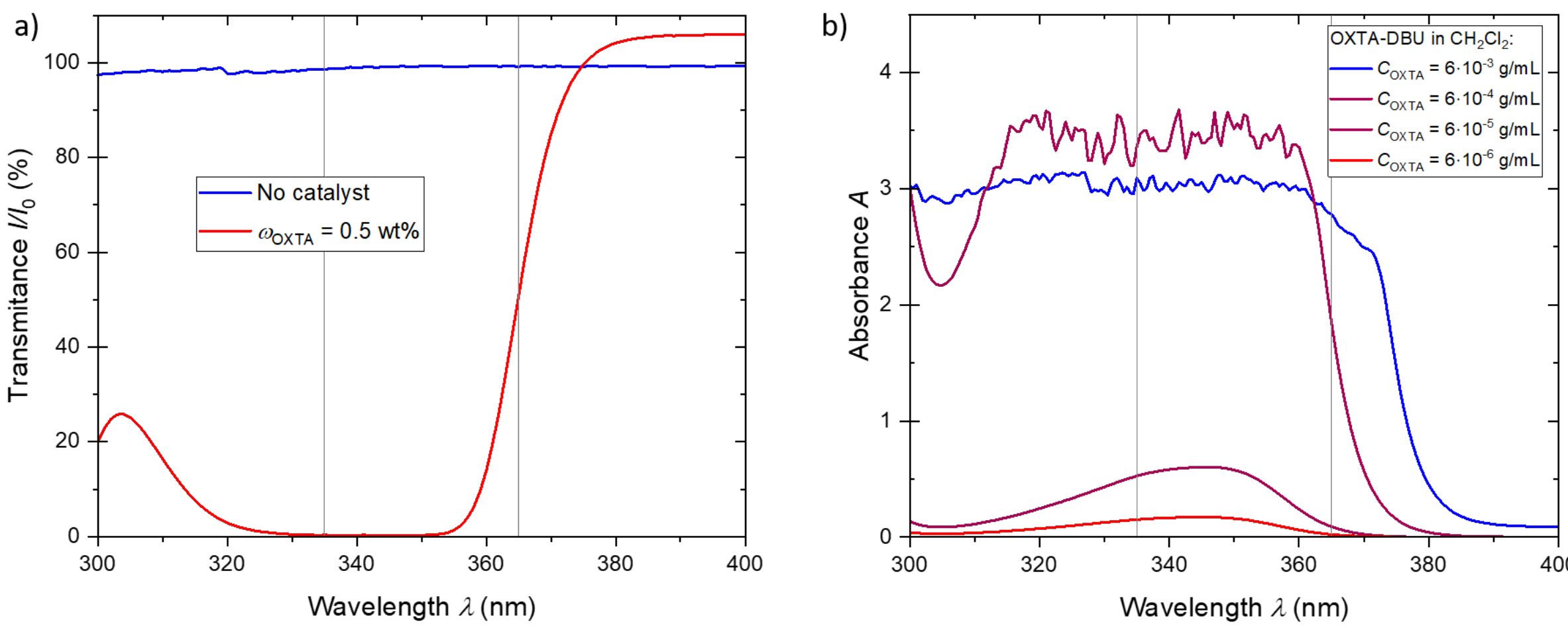

Figure S8: Relative light intensities I/I_0 measured at wavelengths of λ = 335 nm and λ = 365 nm, using a cuvette with an optical pathlength of ℓ = 500 µm and a *Cary 5000 UV-Vis-NIR* Spectrophotometer (*Agilent*) equipped with a *Cary Universal Measurement Accessory* (*Agilent*), as a function of the sensor angle θ, for PU formulations prepared in the same manner as for photorheological measurements and containing ω_{OXTA} = 0.5 wt% and ω_{DBU} = 0 wt% (Table S1). The *Cary Universal Measurement Accessory* was used to displace the sensor around the sample, with θ = 0° corresponding to the sensor position at which it is aligned with the light source and the cuvette holding the sample (the sensor was estimated to cover a span of 20°). These measurements demonstrate in a qualitative manner that, due to the multiple interfaces present in the emulsion constituting the studied PU formulations, most of the incoming light is deviated from its original direction, resulting in measured relative intensities lower than I/I_0 = 2 % at θ = 0°, even in the absence of OXTA-DBU in the system. However, for the catalyst-free sample, the total intensity penetrating across the sample is far from insignificant as the measured relative intensities, although low in value, are spread over a wide area especially at higher angles θ. In addition, we observe once again that the strong absorption coefficient of OXTA-DBU limits the amount of light penetrating through the sample, especially at λ = 335 nm.

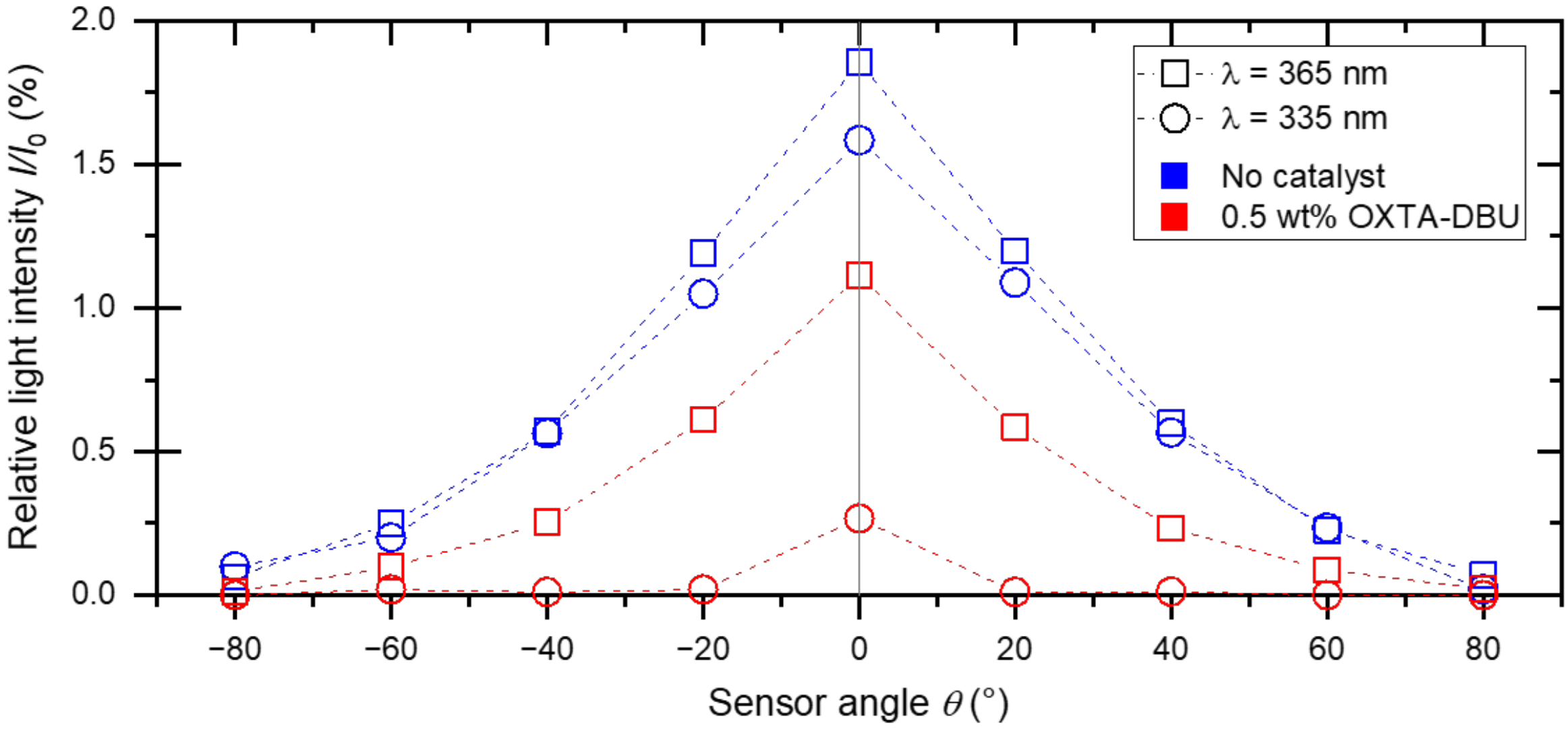

Figure S9: Evolution of the storage modulus G′ and loss modulus G′′ measured via photorheology for formulations with increasing DBU contents (Table S1) in non-irradiated conditions. Results demonstrate that for DBU contents below ω_{DBU} = 0.07 wt% the addition of DBU to the system does not impact *G′* and *G′′* profiles significantly. On the other hand, for $\omega_{DBU} \geq$ 0.1 wt%, *G′* and *G′′* rise more rapidly and cross each other earlier when ω_{DBU} is increased.

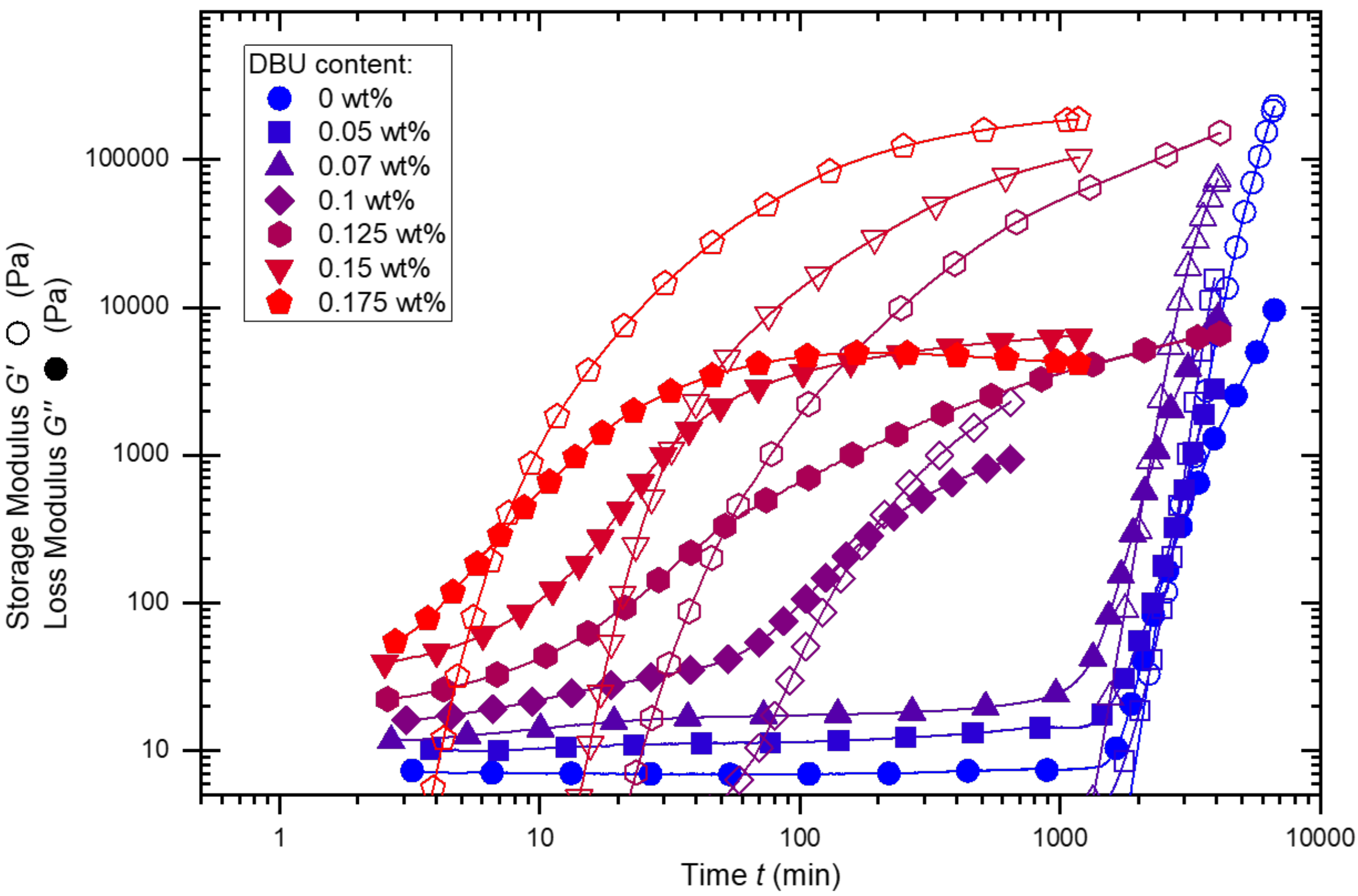

Figure S10: Evolution of the storage modulus *G′* and loss modulus *G′′* measured via photorheology for formulations with DBU content of ω_{DBU} = 0.1 wt% and ω_{DBU} = 0.175 wt% (Table S1) in non-irradiated and irradiated conditions. The results show that, both for ω_{DBU} = 0.1 wt% and ω_{DBU} = 0.175 wt% formulations, the irradiation of the sample has no significant impact on its solidification kinetics.

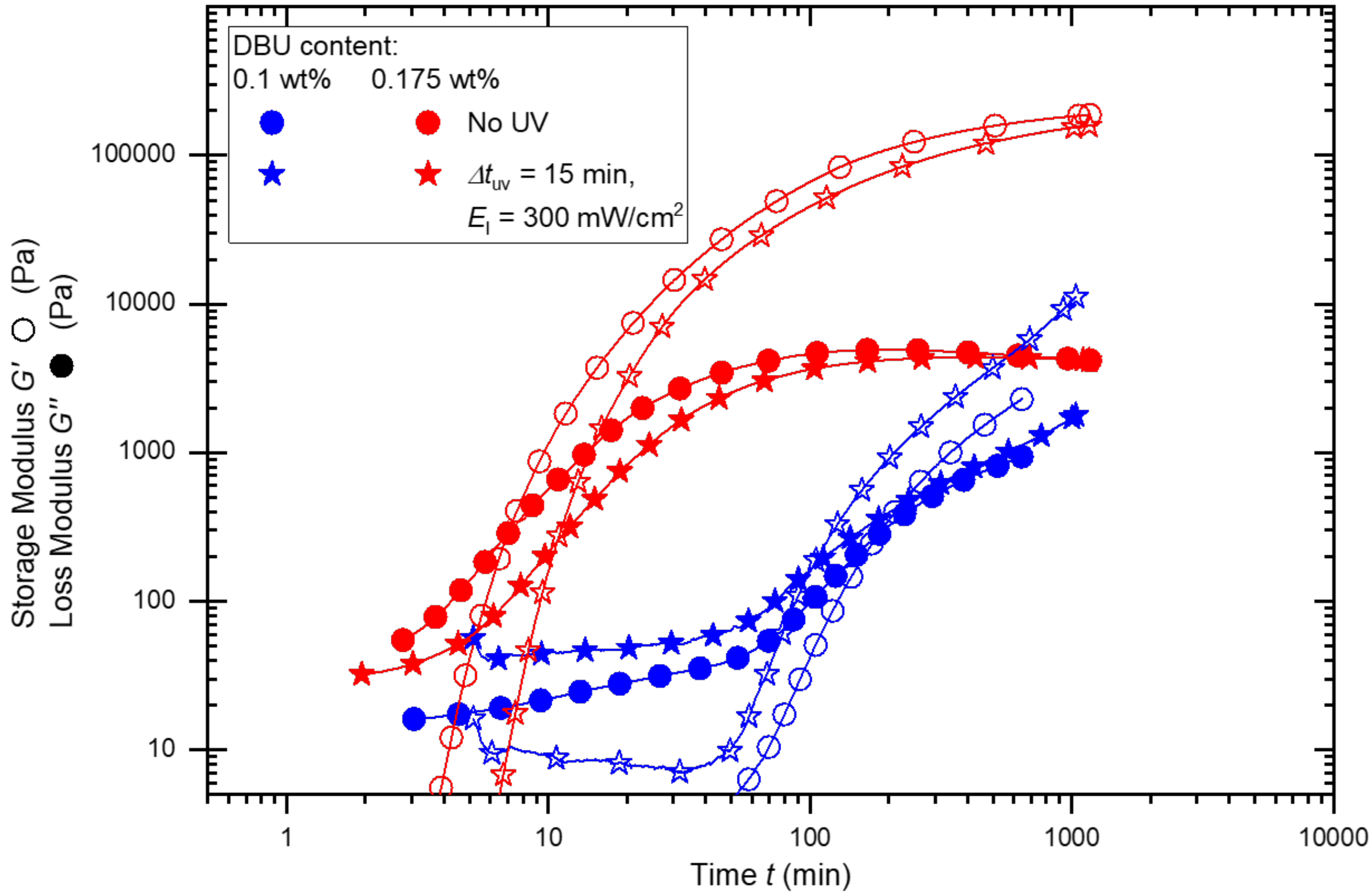

Figure S11: Evolution of the storage modulus *G′* and loss modulus *G′′* measured via photorheology for a formulation with an OXTA-DBU content of ω_{OXTA} = 0.28 wt% (Table S1) (blue circles and triangles) and the hybrid formulations described in Table S2 (other colours), in non-irradiated and irradiated conditions. The results show that, for $\omega_{DBU} \leq 0.05$ wt%, the addition of DBU to the system does not impact *G′* and *G′′* profiles when compared to the ones measured for the formulation containing ω_{OXTA} = 0.28 wt% and ω_{DBU} = 0 wt%. On the other hand, for $\omega_{DBU} \geq 0.083$ wt%, *G′* and *G′′* rise and cross each other significantly faster than for the formulations containing OXTA-DBU alone.

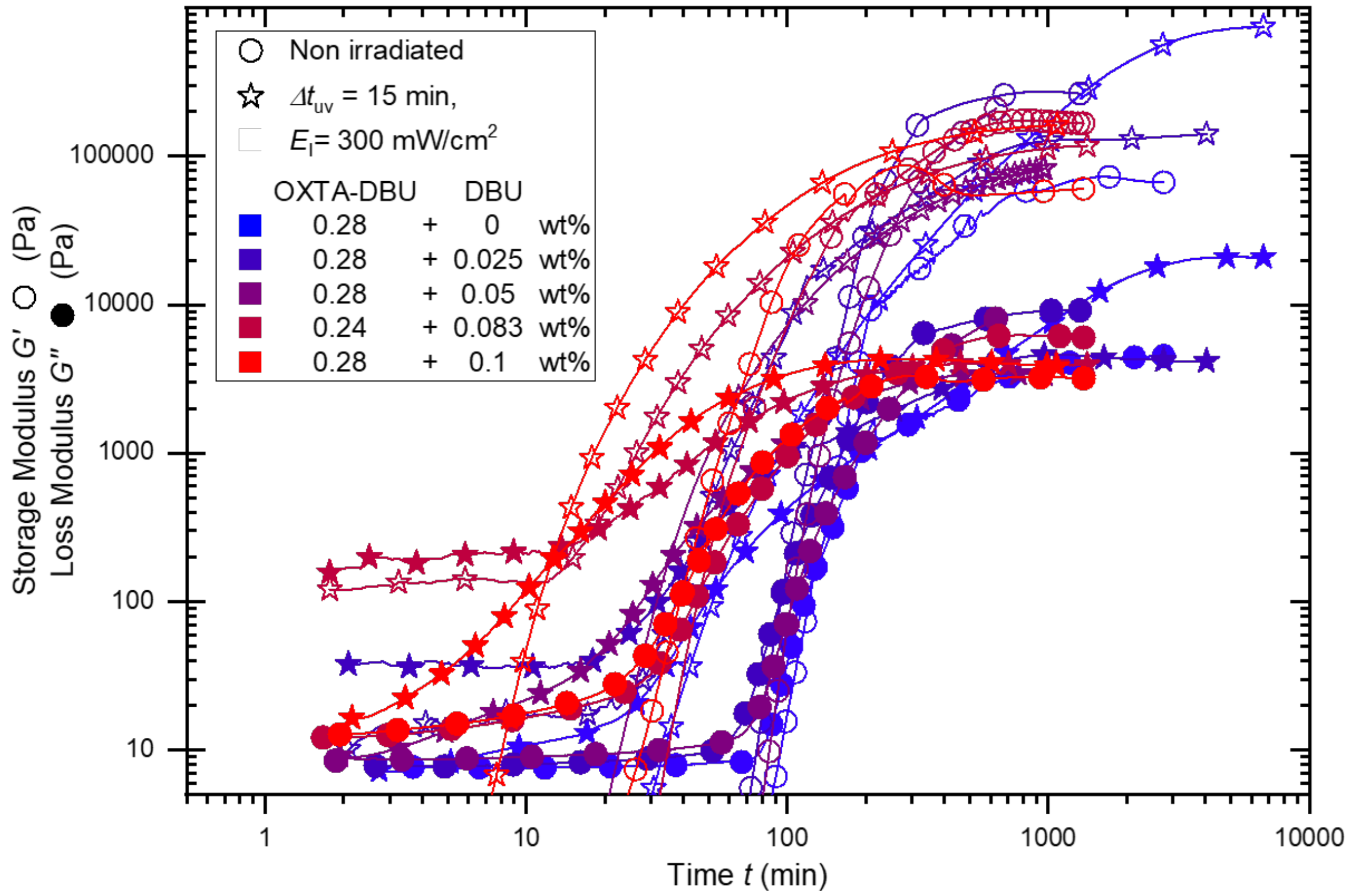

Figure S12: a) 3D reconstructions obtained via microtomography and colour graded according to the local strut thickness calculated using the "Foam and Powder" tool integrated to *VGStudio* for different PU foams. Said PU foams were obtained by letting liquid templates settle for drainage times of Δt_d = 2:15 ± 0:05 min before irradiating them using decreasing irradiation times Δt_{uv}; b) Distribution of the strut thicknesses e_S calculated by *VGStudio* in the same PU foams. We observe in these results that, overall, the struts of foams irradiated for shorter irradiation times Δt_{uv} are thinner on average. This observation demonstrates that shorter irradiation times Δt_{uv} result in slower solidification kinetics, which in turn increase the impact of drainage on the foam morphology, yielding thinner struts.

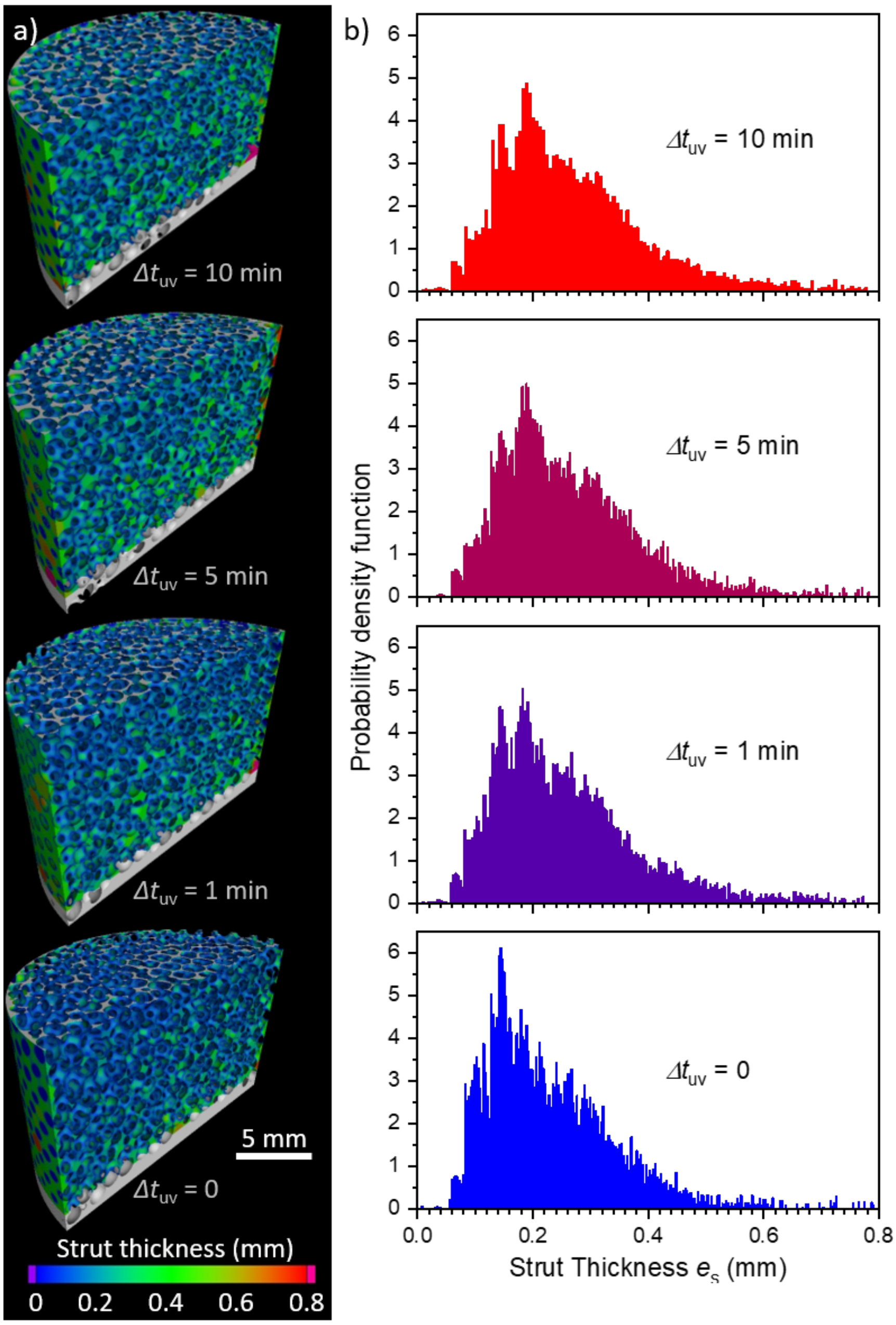

Figure S13: a) 3D reconstructions obtained via microtomography and colour graded according to the local strut thickness calculated using the "Foam and Powder" tool integrated to *VGStudio* for different PU foams. Said PU foams were obtained by letting liquid templates settle for increasing drainage times Δt_d before irradiating them using an irradiation time Δt_{uv} = 5 min; b) Distribution of the strut thicknesses e_S calculated by *VGStudio* in the same PU foams. We observe in these results that, overall, the struts of foams irradiated after longer drainage times Δt_d are thinner on average. This observation demonstrates that the earlier irradiation of a liquid template results in an earlier solidification of the foam, which in turn limits the time during which drainage impacts the foam morphology, leading to the solid foam having thicker struts.

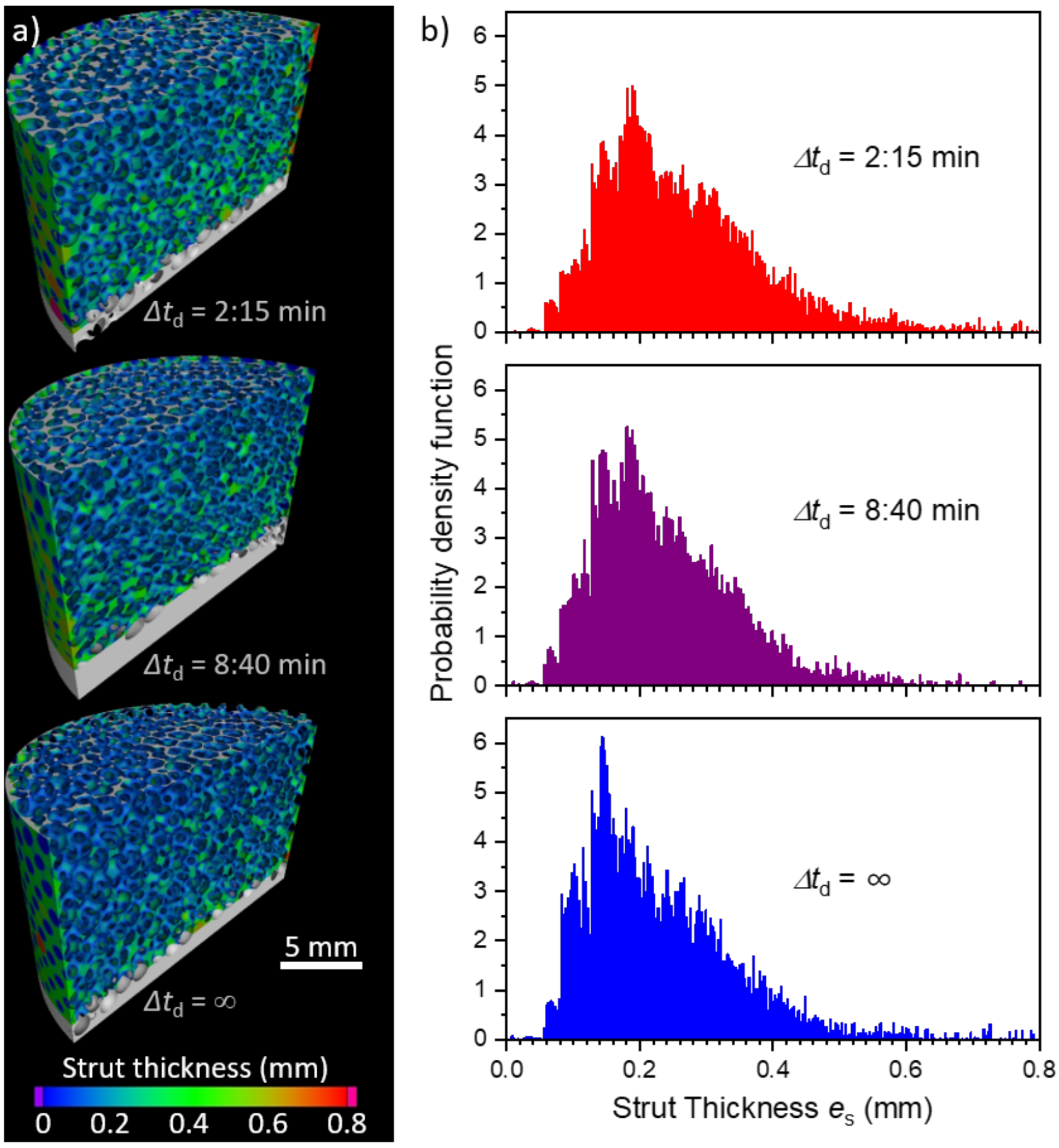

Figure S14: a) Evolution of the solid fraction Φ_S as a function of the vertical position z in four solid PU foams generated from the "Hybrid 4" formulation (Table S2, $\omega_{DBU,eq}$ = 0.2 wt%: ω_{OXTA} = 0.28 wt% and ω_{DBU} = 0.1 wt%), and irradiated after Δt_d = 2:15 ± 0:05 min, using irradiation times of Δt_{uv} = 0, 5, and 10 min and irradiances of E_I = 300 and 600 mW/cm². We can observe that the use of a higher irradiance E_I for the foam irradiation results in an overall higher solid fraction which is a sign of a faster solidification. It is especially interesting to highlight the fact that, for the foam irradiated at E_I = 600 mW/cm² for Δt_{uv} = 5 min, the solidification is even faster than for the foam irradiated at E_I = 300 mW/cm² for Δt_{uv} = 10 min. This demonstrates that the UV dose $UVD = \Delta t_{uv} \cdot E_I$, i.e. the total amount of light shone onto the sample, is not the sole parameter impacting the solidification speed and that the irradiance E_I plays a crucial role, either through a faster activation of the photobase generator or through thermal effects.

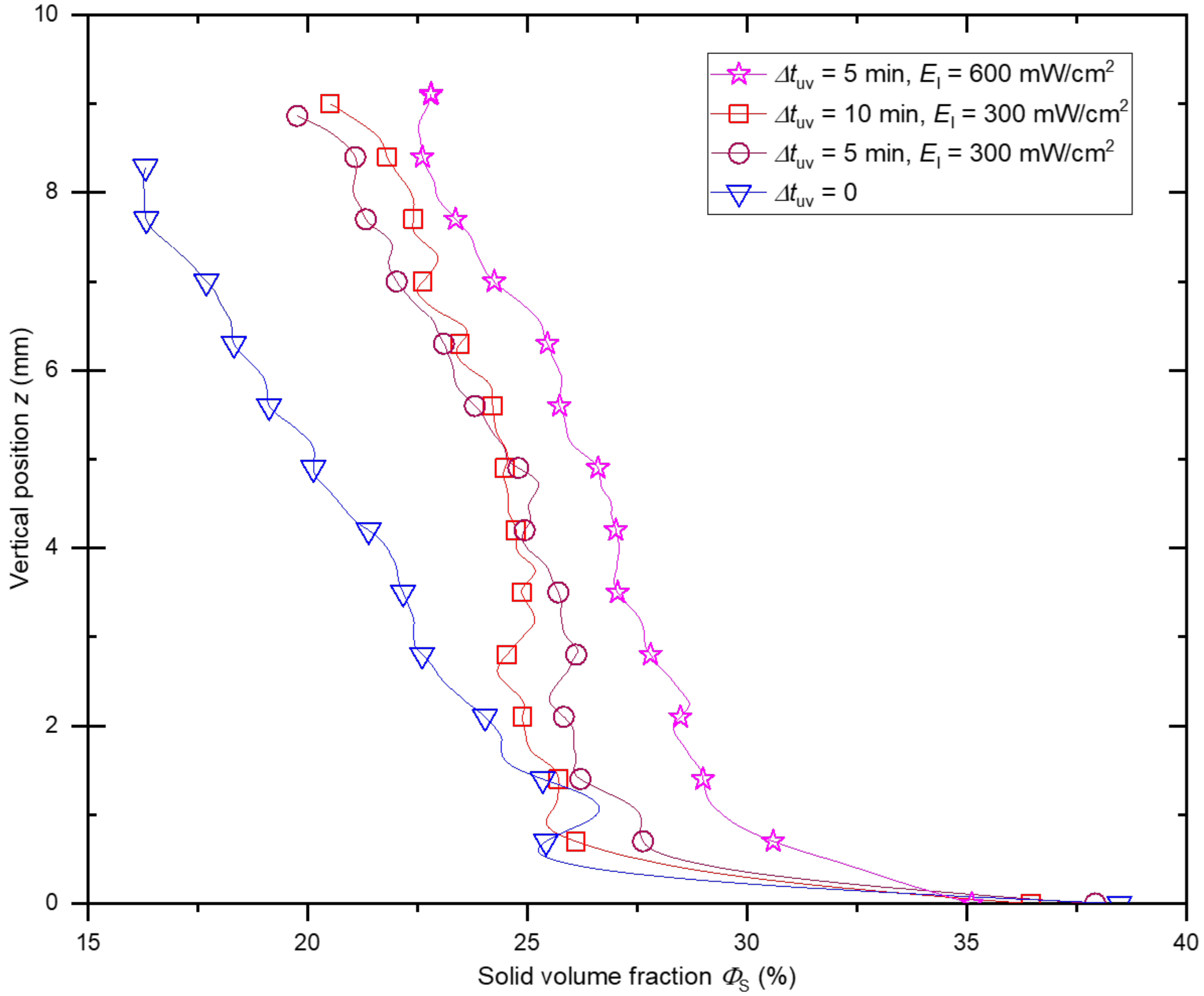

Figure S15: (Left) Evolution of the temperature measured by thermocouples (type K, chromel-alumel) positioned at different depths below the surface of a PU foam generated from the "Hybrid 4" formulation, as a function of time during irradiation (first 15 min) and after irradiation at $E_I \approx 300$ mW/cm^2; (Right) Image of the same foam taken using an IR camera at t = 15 min. This figure demonstrates that, during the irradiation step, the temperature of the foam increases drastically near its top, however this temperature increase becomes much weaker deeper into the foam.

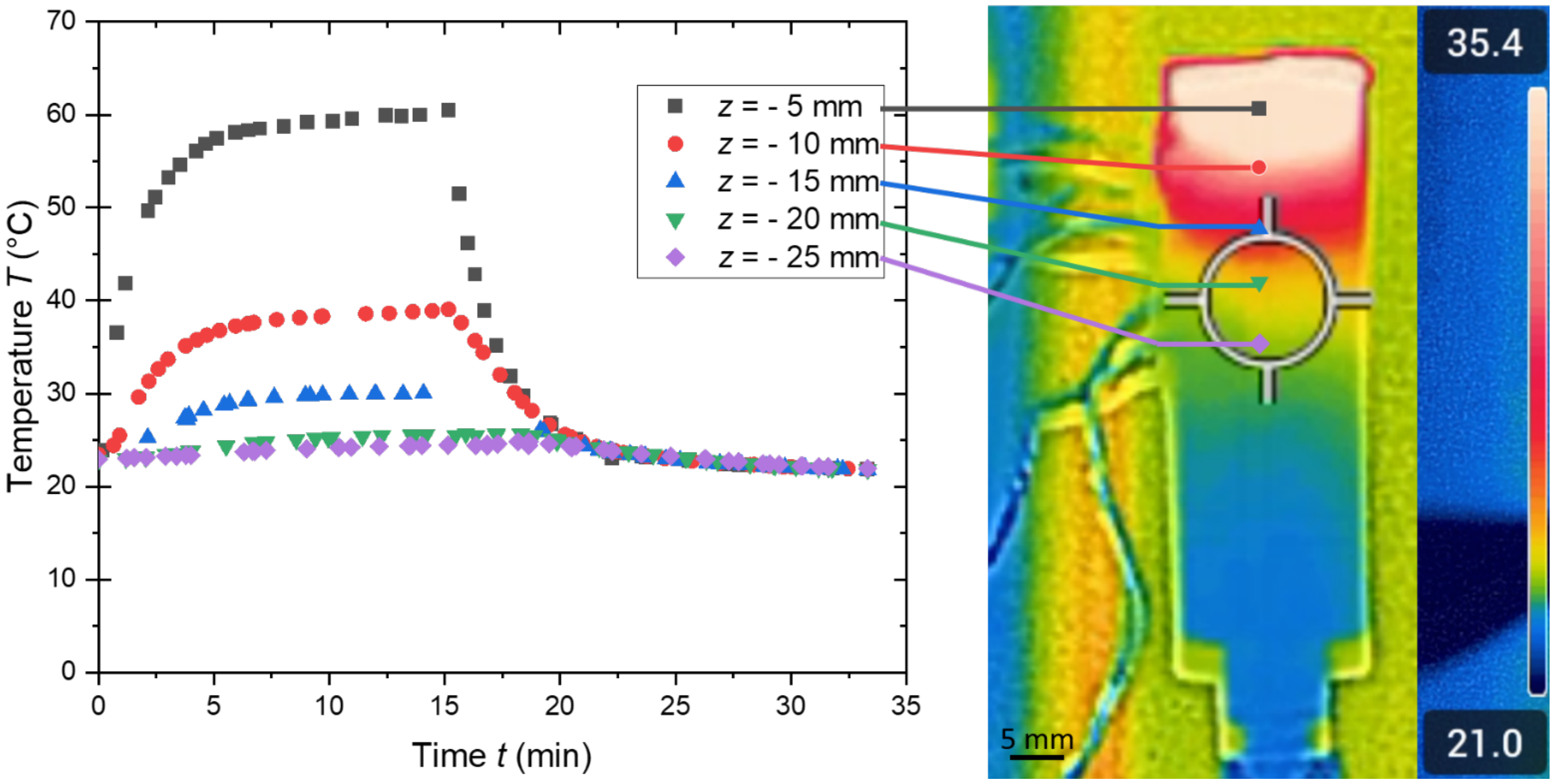

Supplement: Supplementary file 1 [file RA-016-D6RA03376H-s002.pdf]
